# The role of nitric oxide during embryonic wound healing

**DOI:** 10.1186/s12864-019-6147-6

**Published:** 2019-11-06

**Authors:** Pavel Abaffy, Silvie Tomankova, Ravindra Naraine, Mikael Kubista, Radek Sindelka

**Affiliations:** 1 Institute of Biotechnology of the Czech Academy of Sciences – BIOCEV, Prumyslova 595, 252 50 Vestec, Czech Republic; 2 TATAA Biocenter, Odinsgatan 28, 411 03 Göteborg, Sweden

**Keywords:** *Xenopus laevis*, Nitric oxide, Wound healing, Transcriptome, RNA-sequencing, Leptin, AP-1

## Abstract

**Background:**

The study of the mechanisms controlling wound healing is an attractive area within the field of biology, with it having a potentially significant impact on the health sector given the current medical burden associated with healing in the elderly population. Healing is a complex process and includes many steps that are regulated by coding and noncoding RNAs, proteins and other molecules. Nitric oxide (NO) is one of these small molecule regulators and its function has already been associated with inflammation and angiogenesis during adult healing.

**Results:**

Our results showed that NO is also an essential component during embryonic scarless healing and acts via a previously unknown mechanism. NO is mainly produced during the early phase of healing and it is crucial for the expression of genes associated with healing. However, we also observed a late phase of healing, which occurs for several hours after wound closure and takes place under the epidermis and includes tissue remodelling that is dependent on NO. We also found that the NO is associated with multiple cellular metabolic pathways, in particularly the glucose metabolism pathway. This is particular noteworthy as the use of NO donors have already been found to be beneficial for the treatment of chronic healing defects (including those associated with diabetes) and it is possible that its mechanism of action follows those observed during embryonic wound healing.

**Conclusions:**

Our study describes a new role of NO during healing, which may potentially translate to improved therapeutic treatments, especially for individual suffering with problematic healing.

## Background

Wound healing and its regulation is an attractive and rapidly developing field of biology and medicine. The importance for the better understanding of wound healing mechanisms and their regulation is getting more attention because of its relation to the increasing number of ageing people [1]. Defects in wound healing have often been associated with the onset of civilization diseases, where it still remains a burden to the medical system [2, 3]. Therefore, a better comprehension of the wound healing mechanism should lead to the implementation of more effective and cheaper treatments.

The processes of wound healing are very similar amongst different species, ranging from simple organisms like *Drosophila* to more complex mammals like humans [4]. Two types of wound healing, adult and embryonic, have already been identified. Adult wound healing is a much more complex process than embryonic, and leads to troublesome scar formation. The adult wound healing can be divided into four phases: haemostasis, inflammation, proliferation and remodelling [5, 6]. Haemostasis starts immediately after the injury. The main purpose of haemostasis is to avoid blood loss. It involves three processes: blood vessel constriction, a formation of a temporary plug by platelets and clotting (coagulation) of blood at the site of damage [7]. The second phase is called the inflammatory phase and it partially overlaps with haemostasis. In this phase, the immune system is activated and inflammatory cells are recruited from the bloodstream [8]. In humans, haemostasis and inflammatory phases typically last from several hours up to several days. The next phase, proliferation, is characterized by an increase in cell proliferation, which is required for the completion of wound closure. The proliferation phase usually takes several days or even weeks. The final phase is remodelling, when the wound is closed and a new tissue structure beneath is formed including a scar layer which consists of fibrous material deposits [9].

Contrastingly, multicellular embryonic wound healing has only two phases: fast contraction and migration with wound closure [10, 11]. Immediately after injury, the cells at the wound edge produce a high level of calcium, which activates phosphorylation of extracellular signal-regulated kinases (ERK). Calcium release is followed by the formation of an actinomyosin ring and wound tissue contraction [12]. This early phase takes about 30 min and results in about 80% of wound closure [13]. In the second phase of embryonic healing, filopodial protrusions are formed to complete the wound closure [10, 14–17] and *de novo* gene expression of healing specific genes is induced [18]. During this late phase, production of small and biologically active molecules such as reactive oxygen species (ROS) are observed [19–22].

Comparison of adult and embryonic wound healing processes show similarities, but also differences [4]. The most important aspect of healing in embryos is the ability to heal without a scar. Such a phenomenon was described in many animal species including mammalian embryos before the third trimester of pregnancy [23, 24]. This encourages the importance of studies to elucidate regulation and signalling pathways of embryonic wound healing in order to translate then for use in adult wound therapeutic treatments. Several animal models such as *Drosophila*, *Caenorhabditis* and *Danio* have been introduced in the last decade to elucidate various steps of embryonic wound healing [25–28]. In addition, *Xenopus laevis* embryos have become a very popular model for epithelial wound healing studies. Different embryonic developmental stages of the *Xenopus* such as the egg, blastula, gastrula and even later stages have shown relatively similar healing responses including calcium release and actinomyosin ring formation [10, 12, 29–32].

Importance of calcium and ROS production during healing have been shown many times [12, 21, 33–36]. However, only recently the small radical molecule, nitric oxide (NO), have also been implicated as having a role during healing [37, 38]. NO is a gasotransmitter and free radical, that regulates various biological processes. Low levels of NO usually have stimulatory effects on cells during blood pressure regulation [39], proliferation [40], angiogenesis [41] or neurotransmission [42]. In addition, NO has been observed to regulate key processes of adult healing, such as angiogenesis [43], inflammation, cell proliferation, differentiation and apoptosis [44] together with matrix deposition and tissue remodelling [45]. Activity of NO is usually associated with its primary downstream “canonical” pathway. NO is produced by NO synthases (NOS) during the conversion of L-arginine to L-citrulline [46]. The produced NO can then react with the active site of soluble guanylate cyclase (sGC). The activated sGC transforms GTP into cyclic GMP (cGMP). cGMP than activates protein kinase G (PKG, cGMP-dependent protein kinase), which phosphorylates various downstream targets, such as myosin light chains phosphatase responsible for different biological process, such as smooth muscle relaxation [47]. In contrast to physiological low NO level and its activity through the canonical cGMP-dependent pathway, high-level NO acts through cGMP-independent pathway and has instead a detrimental effect on cell viability where it also acts as an antibacterial agent stimulating inflammation [48]. At high concentrations, NO reacts with oxygen radicals and forms aggressive molecules of peroxynitrite, which then nitrosylates nitrosates or nitrates different signalling proteins [49, 50]. For example, this modification leads to a loss of DNA binding capacity of nuclear factor kappa-light-chain-enhancer of activated B cells (NF-κB), reduction in the regulation of transcription [51] or to the inhibition of the activity of c-Jun N-terminal kinase (JNK) which is used to phosphorylate c-Jun [52].

In our study, we found strong NO production in the wounded tissue of embryos at various developmental stages. Early stages of embryos still lack functional immune and blood systems, which are the main components with known NO activity during adult wound healing. We hypothesized that NO is an important factor also during embryonic wound healing. Here we demonstrated the importance and necessity of NO production during early and late phases of embryonic wound healing and suggest a new mechanism of NO activity by regulation of gene expression of key healing signalling pathways.

## Results

### A burst of NO production is a universal response to injury for embryos after the blastula stage

**Fig. 1:**
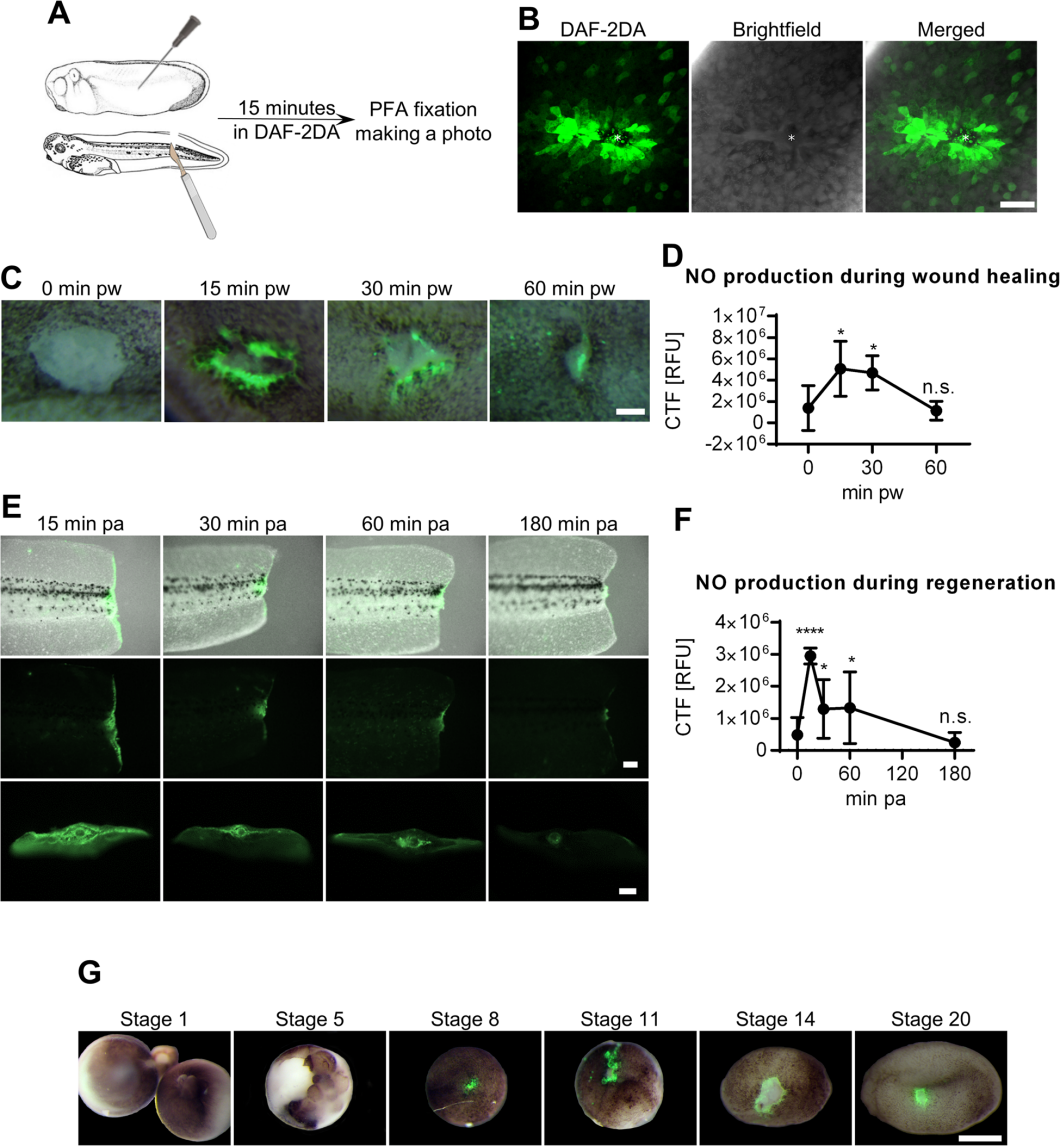
Production of NO during wound healing and regeneration. (**a**) Control embryos at stage 26 were injured using a needle, or tails of tadpoles at stage 41 were amputated and incubated in media with DAF-2DA solution for 15 minutes, fixed and imaged. (**b**) NO is produced in the first two layers of cells around wound edge (Scale bar = 20 μm). (**c**, **d**) NO is produced mainly during first 15 minutes after injury in embryos at stage 26 (Scale bar = 100 μm, five replicates, mean with standard deviation, One-way ANOVA Dunnett’s multiple comparisons test) (**e**, **f**) and after amputation in embryos at stage 41. (Scale bars = 200 μm, three replicates, mean with standard deviation, One-way ANOVA Dunnett’s multiple comparisons test) (**g**) NO is not produced after injury at stage 1 and stage 5, but NO is produced after injury at stage 8 (blastula), stage 11 (gastrula), stage 14 (early neurula) and stage 20 (late neurula) (Scale bar = 500 μm). CTF – corrected total fluorescence, RFU – relative fluorescent unit, pw – post wounding, pa – post amputation **** - *p* < .0001, * - *p* < .05, n.s. - *p* > .05

NO production during wound healing was studied using 5,6-Diaminofluorescein diacetate (DAF-2DA) reporter molecule added to media at tailbud (stage 26) and swimming tadpole (stage 41) stages (Fig. 1a). At the stage 26, the production of NO was observed only within the first two layers of cells at the wound edge (Fig. 1b), with the highest production of NO being observed between 15 and 30 min after injury (Fig. 1c). The NO production increased more than 3.5 times at 15 min post-wounding (pw) compared to the physiological NO level. The NO production decreased at 30 min and returns to the physiological level at 60 min (Fig. 1d). Similar NO production changes were observed after tail amputation (Fig. 1e, f). The highest NO production (more than 6-fold increase) was observed at 15 min post-amputation (pa). The NO production then decreased at 30 and 60 min pa and returned to physiological level at 180 min pa. Moreover, burst of NO production was observed also during the wound healing at stage 8 (blastula), stage 11 (gastrula) and stages 14–20 (neurula). In contrast, NO production was absent during wound healing of earlier developmental stages such as stage 1 (egg) or stage 5 (Fig. 1g).

### NO is crucial for embryonic wound healing

**Fig. 2:**
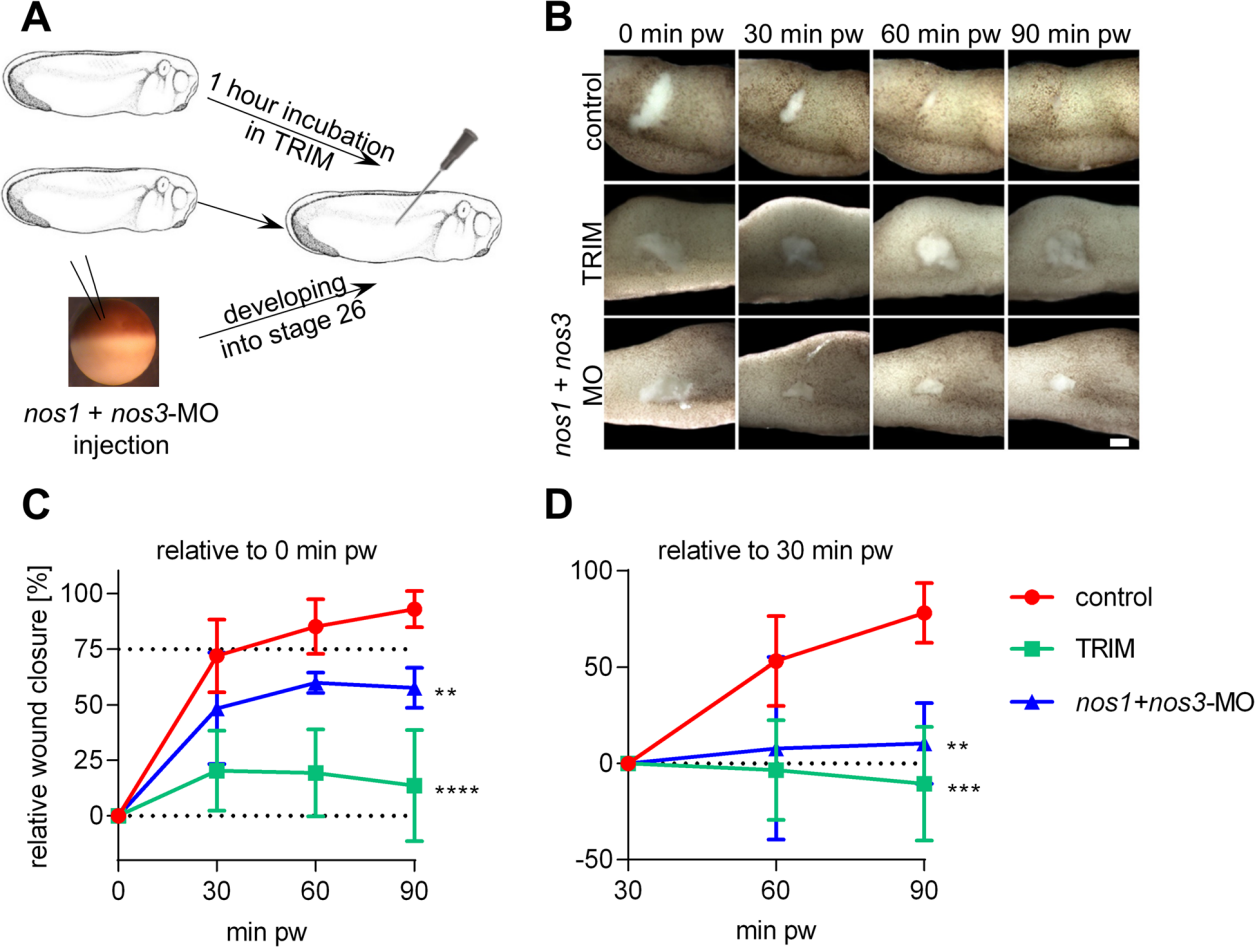
Monitoring of wound closing after inhibition of NO production. (**a**) Control embryos, embryos with inhibited production of NO using TRIM 1 hour before injury and embryos injected with the mixture of *nos1* + *nos3*- MO were injured using a needle (stage 26). (**b**) Wound closing was documented using brightfield imaging on stereomicroscope (Scale bar = 100 μm). (**c**) Relative wound closure was calculated as ratio between the size of the wound in 0 minutes (**d**) or 30 minutes pw (at least three replicates per condition, mean with standard deviation, the statistical difference between the groups is derived from two linear mixed models). pw – post wounding **** - *p* < .0001, *** - *p* < .001, ** - *p* < .01

The importance of NO production during embryonic wound healing was studied using two complementary tools: a NOS chemical inhibitor called TRIM and gene specific knock down of both *nos1* and *nos3*. TRIM treatment simulated acute efficient inhibition of NO production, while *nos1* + *nos3*-MOs injection reflected a chronical decrease of NO production (Fig. 2a). In both cases, inhibition of NO production led to phenotypic defects in the wound healing process (Fig. 2b). In the control embryos, the wound size was reduced to 25% at 30 min pw and it was fully closed at 90 min pw (Fig. 2c). Inhibition of NO production led to significantly slower injury closing. During the first 30 min the wounds closed to only 52% of size (*nos1* + *nos3*-MOs) and of 85% size (TRIM treated) of injury area. Wound closing in the embryos with inhibited NO production was almost stopped after 30 min pw (Fig. 2d). Altogether, results support necessity of NO production especially during early phase of embryonic wound healing.

### Embryonic wound healing is regulated by a modest number of genes

**Fig. 3:**
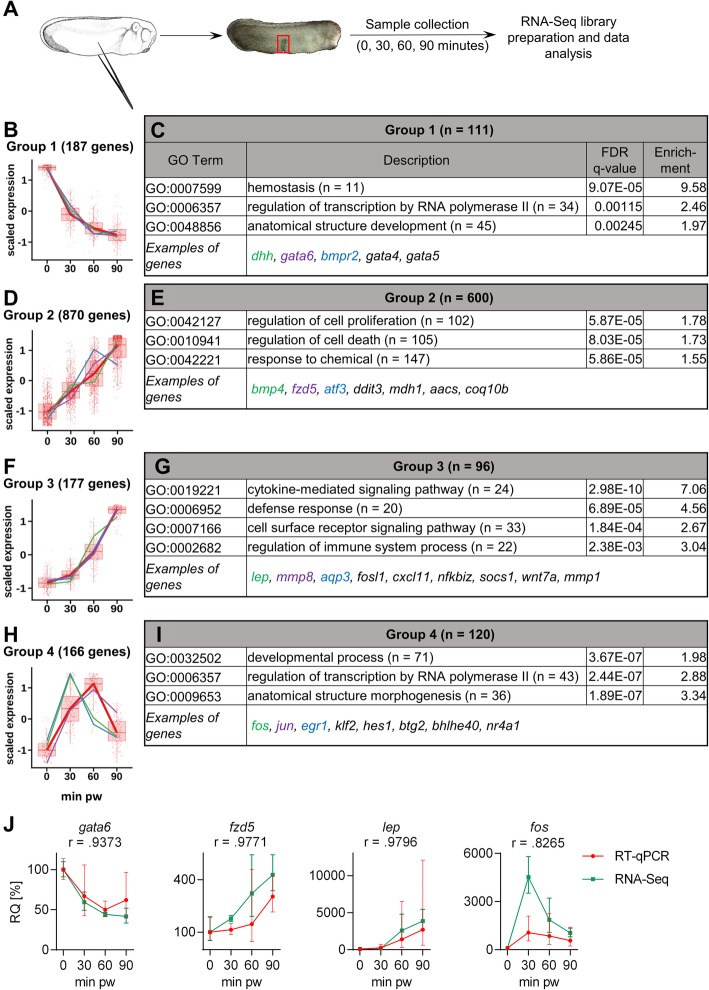
Global gene expression profiles during embryonic wound healing. (**a**) Control embryos at stage 26 were injured using forceps and healing tissues were dissected (only the part marked by red rectangle) and collected for RNA-Seq analysis. (**b**-**i**) DEGs were grouped based on their expression profile relatively to 0 minutes and GO analysis was performed. (**b**, **d**, **f**, **h**) Expression profiles of genes are representative of the log transformed data, average gene expression is shown in red and expression of three representative genes are shown in green, purple and blue. (**c**, **e**, **g**, **i**) Genes, which have an annotation and human homolog, were used for GO analysis. Numbers of analysed genes are in the table together with the representative GO terms for each group. (**j**) Validation of RNA-Seq data by RT-qPCR using representative genes from each Group was performed using RT-qPCR and the Pearson r correlation coefficient was calculated from the geometric mean values. (RNA-Seq – three replicates, RT-qPCR – six replicates, geometric mean with geometric standard deviation). DEGs – differentially expressed genes, pw – post wounding

The transcriptome analysis of the healing tissue at the key phases (uninjured tissue (time 0), and 30, 60, 90 min pw) using tailbud (stage 26) embryos was performed (Fig. 3a, Additional file 1: File S1). In total 23,609 genes were found to be expressed in the dissected healing area. A total of 2128 genes (9.0%) were identified as differentially expressed genes (DEGs) during 90 min of embryonic wound healing. 6% of these DEGs (134 genes) (example: *lep*, *fosl1*, *aqp3*) were found to be specific for healing (absent in uninjured tissue but expressed during healing). DEGs were clustered into four groups based on their temporal expression profiles (Fig. 3b-i). Number of genes for Gene Ontology (GO) analysis was reduced to include only well annotated genes with a known human homolog. Several interesting genes for each group are presented based on their importance for the regulation of healing and development as derived from published literature. Downregulated genes during wound healing were clustered in Group 1 (Fig. 3b). This group contained 187 genes (111 for GO analysis) such as *dhh*, *gata6* and *bmpr2*, which are mainly responsible for processes involved in the regulation of developmental control (Fig. 3c, Additional file 2: Figure S1A). Members of Group 2 showed a continuous increase of gene expression during the first 90 min of healing (Fig. 3d). This group comprised of 870 genes (600 for GO analysis), with their GO terms indicating that they are responsible for regulation of cell proliferation, cell death and response to chemical stimuli (Fig. 3e, Additional file 2: Figure S1B). The Group 3 consisted of 177 genes (96 for GO analysis), whose expression started 30 min pw (Fig. 3f). These genes are associated with cytokine-mediated signalling pathways and with the defence/immune response (Fig. 3g, Additional file 2: Figure S1C). The Group 4 represents 166 genes (120 for GO analysis) that were expressed strongly between 30 and 60 min with minimal expression before and after this period (Fig. 3h). Interestingly, most of the genes from the Group 4 regulate transcription, with many of them being associated with the AP-1 transcription pathway (Fig. 3i, Additional file 2: Figure S1D). The remaining 728 DEGs clustered into two additional groups (Additional file 1: File S1). However, they were excluded from further analysis due to the low reproducibility (high variability between experiments) and low expression changes (absolute value of log2FoldChange < 1 for comparison between 0 min pw and any time point for most of the genes) which suggested their potentially minimal effect during healing.

Using independent experimental setups, the temporal profile for one gene from each major cluster group was analysed using RT-qPCR. The profile results correlated well with those obtained from the RNA-Seq (Fig. 3j).

### NO is important for the regulation of gene expression during embryonic wound healing

**Fig. 4:**
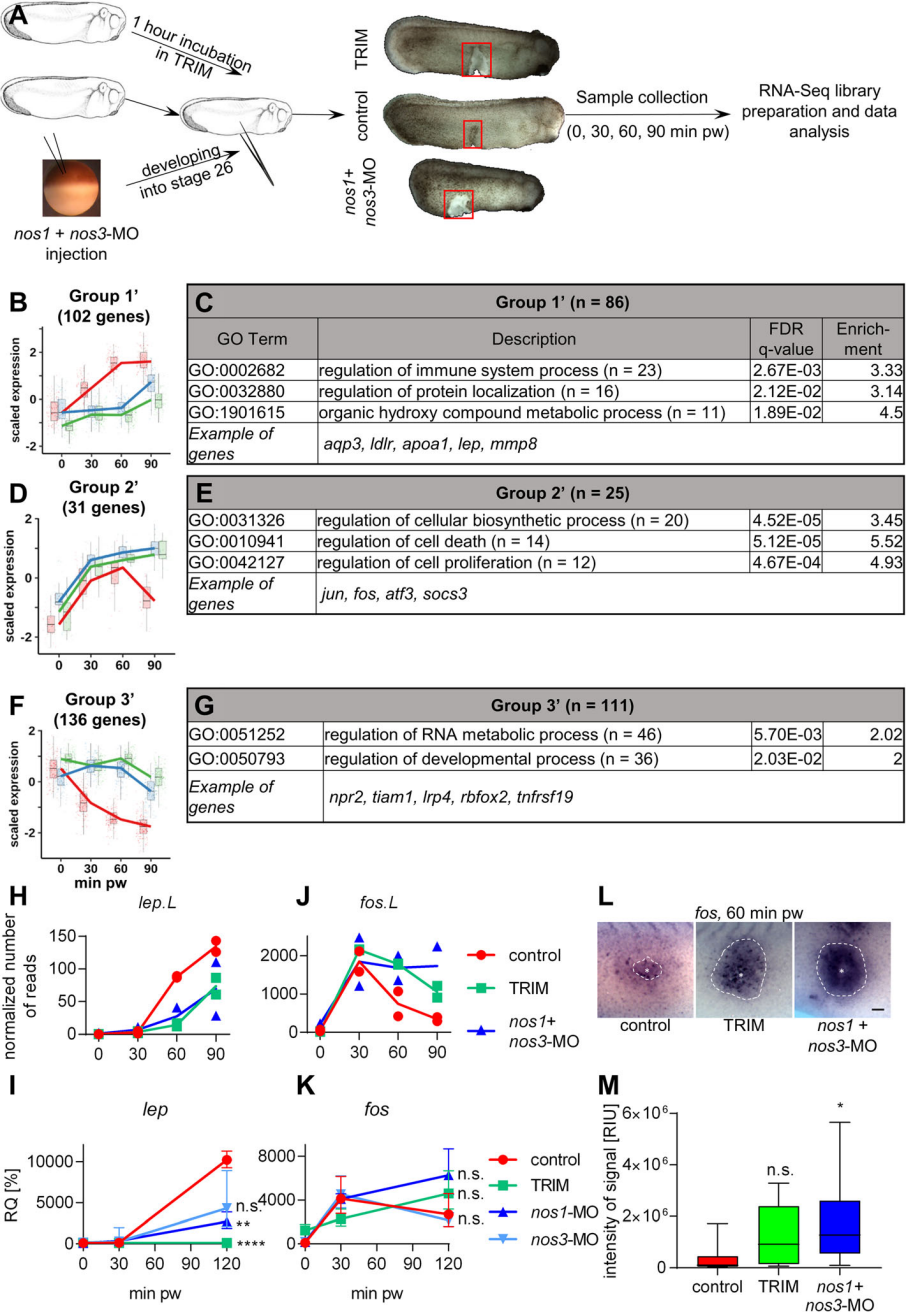
Changes in gene expression during wound healing after inhibition of NO production. (**a**) Graphical description of RNA-Seq experiment comparing control and NO inhibited embryonic wound healing. Only the part marked by red rectangle was collected and used for RNA isolation and sequencing. (**b**-**g**) DEGs, which were identified in RNA-Seq, were grouped based on their expression profile relatively to 0 minutes and GO analysis was performed. (**b**, **d**, **f**) Expression profiles of genes are representative of the z-score of the regularized log transformation of the normalized counts. (**c**, **e**, **g**) Genes with annotation and human homolog were used for GO analysis. Numbers of analysed genes are in the table together with the representative GO terms for each group. (**h**) RNA-Seq result of *lep* expression was verified (**i**) using RT-qPCR, separately for *nos1*-MO and *nos3*-MO. (**j**) Similarly, RNA-Seq result of *fos* expression was verified using (**k**) RT-qPCR (data are normalized to 0 minutes pw in controls, three replicates, geometric mean with geometric standard deviation, two-sided t-test from log2 values of relative expression between inhibited samples and control in 120 minutes pw), and (**l**) in situ hybridization. Site of injury is marked with a star and the signal where *fos* is expressed is circled by dot line (Scale bar = 100 μm) (M) Intensity of blue signal around site of injury were measured (one-way Anova, Dunnett’s multiple comparisons test, minimum 8 replicates). **** - *p* < .0001, ** - *p* < .01, * - *p* < .05, n.s. - *p* > .05 DEGs – differentially expressed genes, pw – post wounding, RIU – relative intensity unit

RNA-Seq comparison between control and NO inhibited embryos (mixture of *nos1* + *nos3*-MO and chemical inhibitor TRIM) was performed using the same experimental workflow as above (Fig. 4a, Additional file 3: File S2). A total of 269 genes showed contrasting expression profiles. Clustering based on expression profiles across the two conditions, divided genes into three groups labelled with an apostrophe (′) (Fig. 4b-g).

Group 1′ consisted of 102 genes (86 for GO analysis), whose expression was increased in control embryos, but their expression was stable/absent after NO inhibition (Fig. 4b). Based on the GO analysis, these genes are mainly responsible for the regulation of immune system response (Fig. 4c, Additional file 4: Figure S2A). Thirty-one genes (25 for GO analysis) clustered in Group 2′ and their expression was similar until 60 min pw between control and inhibited samples. After that their expression was quickly decreased in control embryos, but continued to increase in the inhibited embryos (Fig. 4d). This group comprised of GO terms related to transcription regulation important for cell proliferation, differentiation and death (Fig. 4e, Additional file 4: Figure S2B). Group 3′ contained 136 genes (111 for GO analysis), whose expression was downregulated in the control embryos, but was not changed after NO inhibition (Fig. 4f). GO analysis identified mainly regulation of developmental and metabolic processes (Fig. 4g, Additional file 4: Figure S2C).

Two interesting genes from Group 1′ and Group 2′, *lep* and *fos* respectively*,* which had minimal expression within the uninjured tissue but fast activation following injury, and also a strong dependence on NO production were selected for detailed analysis. The results from RNA-Seq (Fig. 4h, j) were verified in detail using RT-qPCR (Fig. 4i, k) and *in situ* hybridization (Fig. 4 l, m) using independent experimental samples. Expression of *fos* was dramatically increased after injury in control embryos with peak at 30 min pw. In contrast, NO inhibited embryos showed a stable or a slightly increased *fos* level between 30 to 90 min. *In situ* hybridization of *fos* showed minimal signal at 60 min pw in control embryos, but a strong enrichment in both TRIM and MOs inhibited embryos at that time (Fig. 4 l, m). The *lep* gene was undetectable even by RT-qPCR in uninjured embryos and it was not expressed during 30 min pw. Its expression appeared at 60 min pw and continuously increased. NO inhibition led to significant reduction of *lep* expression (Fig. 4i, k).

### Changes of tissue morphology at the wound edge after NO inhibition

**Fig. 5:**
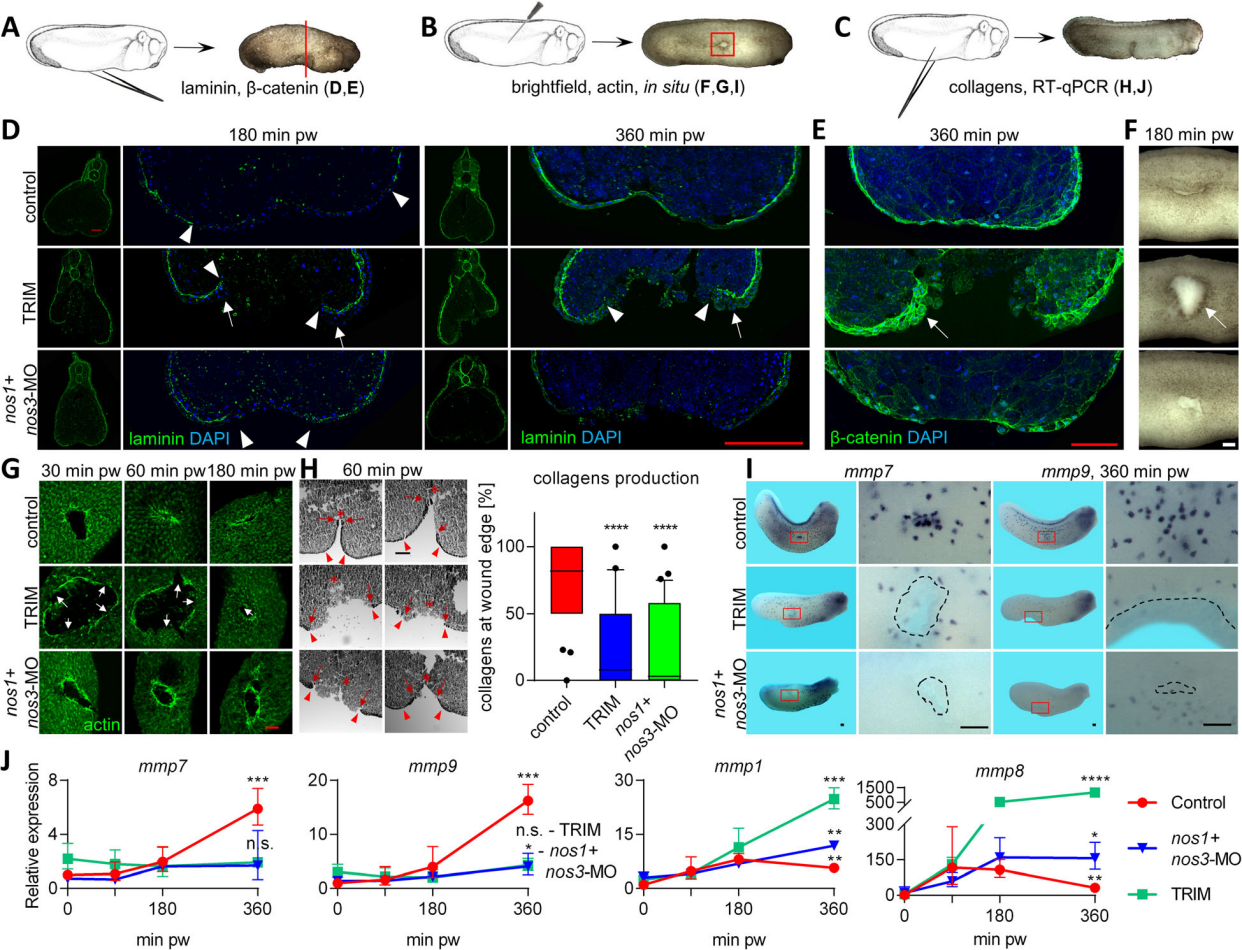
Monitoring of phenotype changes during wound healing in embryos with inhibited NO production. (**a**, **b**, **c**) Control embryos, embryos with inhibited production of NO using TRIM 1 hour before injury and embryos injected with mixture of *nos1* + *nos3*- MO were injured at stage 26 using forceps or needle in the middle and ventral side. (**d**) Laminin layer was visualized at 180 minutes and 360 minutes pw and ends of the laminin layer are marked by a triangle. Formation of “blob” in TRIM embryos is marked by arrow (Scale bar = 100 μm). (**e**) Staining of β- catenin 360 minutes pw (Scale bar = 100 μm). (**f**) Brightfiled image of wound site in 180 minutes pw (Scale bar = 100 μm). (**b**, **g**) Actin at 30, 60 and 180 minutes pw visualized using green fluorescent phalloidin. Breaks in actin layer are marked by arrow (Scale bar = 100 μm). (*c*, *h*) Collagen staining at 60 minutes pw. The beginning of the wound is marked by a red triangle. A red arrow marks the end of the collagen layer, while the end of the wound site is marked by a red star. (Scale bar = 100 μm, measurement of coverage of collagen in wound was made from at least six embryos per condition and at least five slices per embryo, one-way anova, Dunnett’s multiple comparisons test). (**i**) Spatial expression of two matrix metalloproteinases *mmp7* and *mmp9* was visualized by in situ hybridization in time 360 minutes pw (Scale bars = 500 μm). (**j**) RT-qPCR comparison of temporal expression profiles of *mmp1*, *mmp8*, *mmp7* and *mmp9* (data are normalized to 0 minutes pw in controls, three replicates, geometric mean with geometric standard deviation, two-sided ttest from log2 values of relative expression between 360 minutes and 0 minutes). **** - *p* < .0001, *** - *p* < .001, ** - *p* < .01, n.s. - > .05 pw – post wounding

Immunohistochemistry using morphology markers such as actin (cell shape), β-catenin (cell shape) and laminin (basement membrane) was performed to reveal morphological changes at the wound edges of control and NO inhibited embryos (Fig. 5). Laminin staining experiments were performed at 180 and 360 min pw. Control embryos showed discontinuous laminin staining in the wound site 180 min after injury and complete restoration at 360 min pw. Acute inhibition of NO production using TRIM showed minimal laminin production in wound site at 180 and 360 min pw. As expected, acute inhibition had no effect on the laminin layer in the tissue behind the wound edge. Interestingly, usage of *nos1* + *nos3*-MO led to different effects than inhibition using TRIM. In general, the laminin layer was weaker in treated embryos in comparison with the controls even in uninjured tissue and there were no signs of the basement membrane reformation at 360 min pw (Fig. 5d). β-catenin staining revealed changes in cell migration in NO inhibited embryos resulting in accumulation on the wound edge. Especially in TRIM treated embryos, cells responsible for wound closure formed clumps of cell aggregates or “cell blobs” at the wound edges, potentially preventing wound closure (Fig. 5e). These accumulated cells were observed as a dark edge around the wound, which was observable also in the brightfield images of healing embryos (Fig. 5f).

A well-described process during embryonic wound healing is the formation of an actinomyosin ring around the wound and it was studied using phalloidin for staining of actin (Fig. 5g). Acute NO inhibition by TRIM treatment led to decreased formation of actin ring around the wound edge with many breaks within the actin layer. In addition, the cell morphology at the wound edge of TRIM treated embryos was also abnormal (Additional file 5: Figure S3A). Chronic NO inhibition using MOs resulted in a different phenotype. Actin was produced at a higher level around the wound edge and formed a complex structure inside of the injured MO embryos (Additional file 5: Figure S3B).

Collagen synthesis and its correct deposition is also an important step during wound healing. Previous research have already shown that NO is required for collagen synthesis [53, 54]. Masson’s trichrome staining was performed to compare collagen production between control and NO inhibited embryos. Collagen layer around wound edge was studied at 60 min pw. Collagen covered 82% of wound surface in control embryos compared to only 8% in TRIM treated embryos and 3% in NO inhibited embryos (Fig. 5h, i).

Defects of basement membrane formation and collagen synthesis as showed in Fig. 5d and h respectively, are tightly connected with the production of matrix metalloproteinases (MMPs) enzymes that are responsible for tissue remodelling. The spatial and temporal expression analyses of four (*mmp1*, *mmp7*, *mmp8*, *mmp9*) of the most interesting MMPs (based on our RNA-Seq data) were performed during middle and late phases of wound healing (Fig. 5i, j). The genes *mmp7* and *mmp9* are also markers for migrating myeloid progenitors. Additionally, *mmp1* and *mmp8* are known regulators of cell migration during wound healing. Migration of cells expressing *mmp7* and *mmp9* to the wound site were observed at 360 min pw in control embryos. Inhibition of NO production led to a reduction in the number of these cells and also a retardation of their migration (Fig. 5i). RT-qPCR expression profiles revealed an approximately 6-fold and 16-fold increase of *mmp7* and *mmp9* respectively at 360 min pw in control embryos compared to no gene expression changes in NO inhibited embryos (Fig. 5j). RT-qPCR analysis of *mmp1* and *mmp8* showed an opposite result. The *mmp1* and *mmp8* expressions increased during 90 min pw in both the control and treated embryos. However, gene expression at later time points showed a difference between the control and NO inhibited embryos. Whereas *mmp1* and *mmp8* expression started to decrease after 90 min, their levels in NO inhibited embryos continued to increase (Fig. 5j).

### Leptin is a downstream target of NO signalling during the healing

**Fig. 6:**
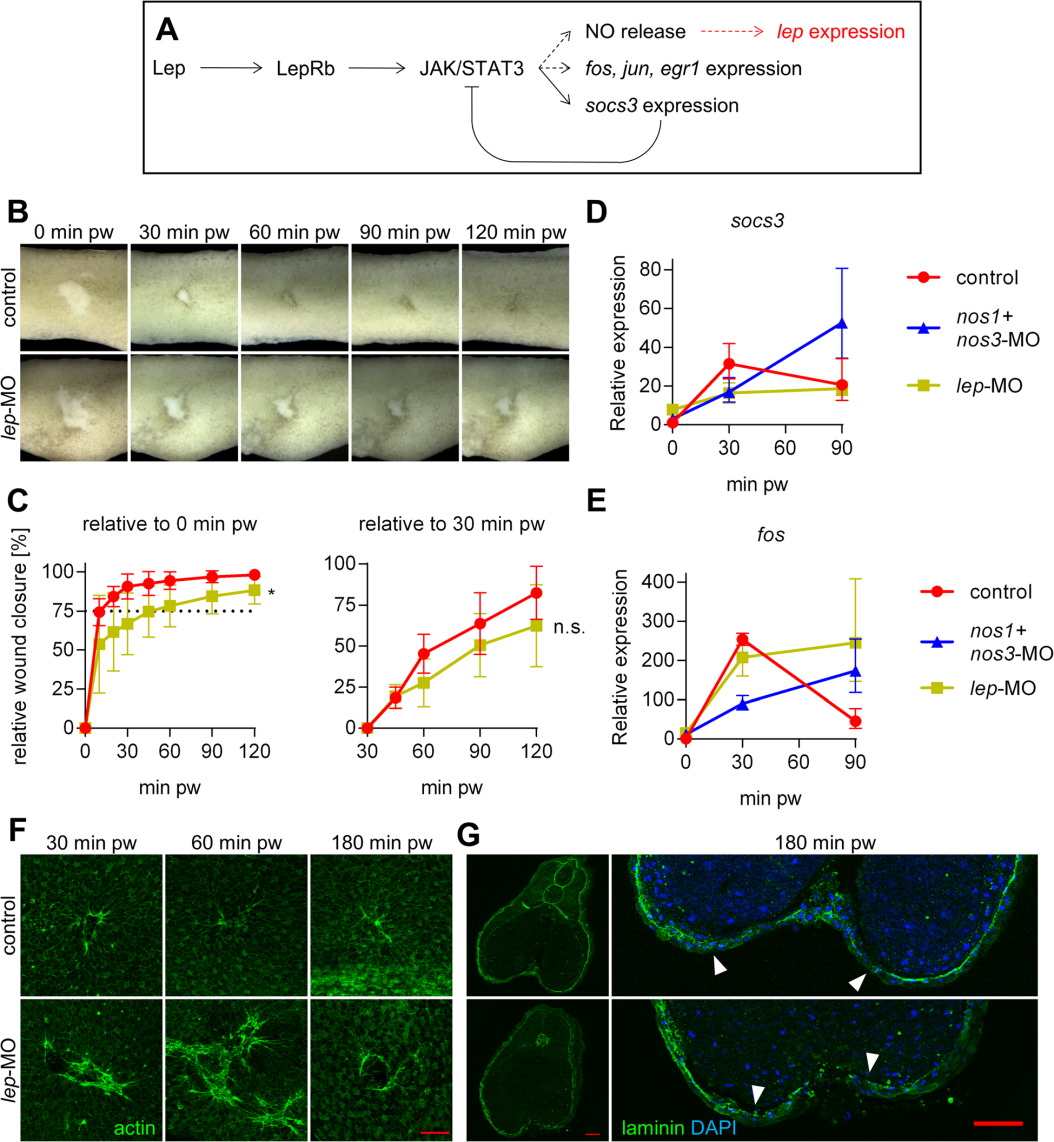
Monitoring of processes during wound healing after inhibition of lep expression. (**a**) In general, Lep is described as an activator of NO release, but inhibition of NO production leads to decreased expression of lep during wound healing. (**b**) Wound closing was documented using brightfield imaging on stereomicroscope. (**c**) Relative wound closure was calculated as the ratio between the size of the wound at 0 minute pw and 30 minutes pw (at least four replicates per condition, mean with standard deviation, the statistical difference between the groups is derived from two linear mixed models). (**d**) RT-qPCR comparison of temporal expression profiles of *socs3* and (**e**) *fos* (data are normalized to 0 minutes pw in controls, three replicates, geometric mean with geometric standard deviation). (**f**) Actin at 30, 60 and 180 minutes pw visualized using green fluorescent phalloidin (Scale bar = 100 μm). (**g**) Laminin layer was visualized at 180 minutes pw and ends of the laminin layer are marked by a triangle (Scale bar = 100 μm). * - *p* < .05, n.s. – *p* > .05 pw – post wounding

Leptin is known as an activator of NO release [55], but RNA-Seq and RT-qPCR comparison between control and NO inhibited embryos revealed *lep* to be downregulated in NO inhibited wound healing (Fig. 6a, Fig. 4h). Importance of *lep* for wound healing was analysed. Usage of *lep*-MO led to a decrease in the speed of wound closure (Fig. 6b, c). Expression of *socs3* is usually measured to monitor the activity of Lep. We confirmed, that usage of *lep*-MO led to no changes of expression of *socs3* during wound healing (Fig. 6d). Control embryos showed an increased expression of *socs3* during the first 30 min pw followed by a decreased expression during the middle phase. However, inhibition of NO production using MOs led to increasing expression of *socs3* during the studied 90 min of embryonic wound healing. Expression of *fos* was similar between both *lep*-MO and *nos1 + nos3*-MO (Fig. 6e). To verify the impact of *lep* for wound healing process, the immunohistochemistry analysis of actin and laminin was performed. Comparison of actin formation at the wound edge showed that *lep* loss-of-function led to extreme formation of actin during first 60 min pw (Fig. 6f) and showed minimal laminin production in wound site at 180 min pw (Fig. 6g).

## Discussion

**Fig. 7:**
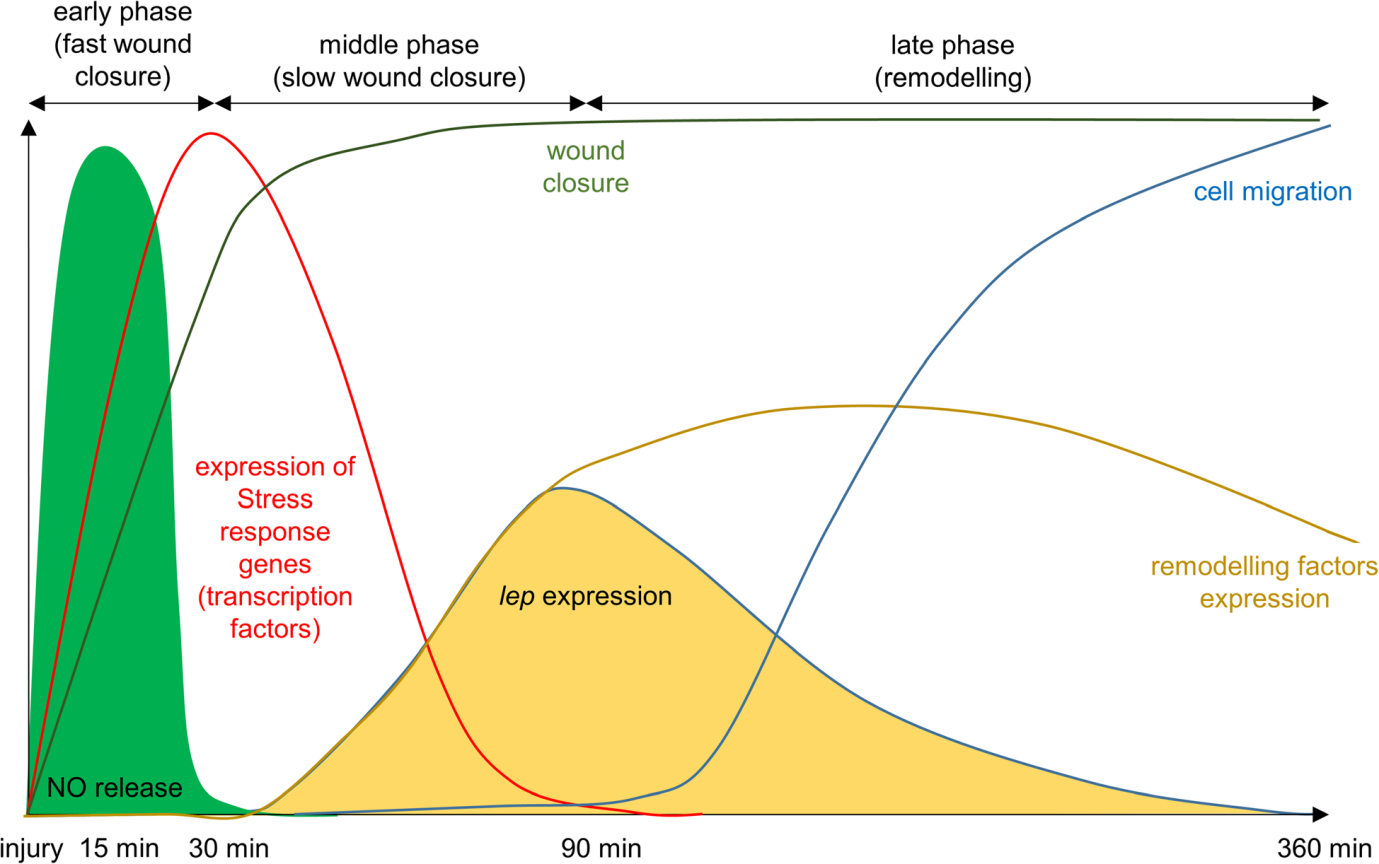
Interpretation of our results – description of processes during embryonic wound healing. NO release appears very early after injury. The level of NO is the highest at 15 minutes pw and the physiological level is restored at 30 minutes pw (early phase of healing). De novo expression of injury response genes starts shortly after injury and the level of expression is the highest 30 minutes pw. Level of expression of injury response genes is restored to physiological level 90 minutes pw. De novo expression of interesting NO dependent candidate *lep* and remodelling factors starts around 30 minutes pw. At the same time around 80 % of injury is already closed (middle phase of healing). The injury is closed 90 minutes pw and remodelling phase is initiated. Cell migration appears and the expression of remodelling factors changes (late phase of healing)

Embryonic wound healing, which results in a scar-less wound closure, is a fascinating biological phenomenon. However, very little is still known about the molecular mechanism that regulate this process. In this research, we utilized the popular model *Xenopus laevis* embryos to reveal the different healing phases and their important genes [10, 28, 56, 57]. Embryonic healing is composed of the early phase which lasts less than 30 min and results in the near completion of wound closure. The following middle phase takes place between 30 and 90 min and ends with the wound site completely closed by filopodia/lamellipodia activity [10]. In our study, we described an additional late healing phase during which the tissue under the wound site is remodelled and could take several hours to complete (Fig. 7).

The important difference between embryonic and adult wound healing is the presence and activity of the immune/inflammation system. Inflammation response has been suggested many times as the key component that results in scaring during adult wound healing [58, 59]. However, recent studies also claim that inflammation is required for embryonic healing and that the mechanism is more complicated [60]. Inflammation is usually characterized by an abnormal production of small radical molecules, which serve as an antimicrobial agent around the wound. ROS are among the most studied molecules during embryonic and adult wound healing [20, 61, 62]. However, the wound healing can also be regulated by other small gas molecules (also called gasotransmitters) such as carbon monoxide (CO) [63, 64], hydrogen sulphide (H_2_S) [65, 66] and NO. Surprisingly, in recent studies, NO received very little attention compared to the ROS. NO is usually connected with angiogenesis and inflammation [67] during adult wound healing [68–70] or with extracellular matrix modifications [38]. In our study, we analysed the role of NO during wound healing at the developmental stage 26. This is an ideal stage for the analysing of embryonic wound healing as it represents the embryonic transitional period between partially differentiated cells of the gastrula stage and the later complex tissues, including blood and immune cells that are already formed during later stages.

### The early phase of healing (0–30 min after injury)

The first NO production connected with healing was detected shortly after injury and its level peaked at 15 min (Fig. 1c-e). The level of NO was abnormally high compared to the physiological level and we estimate that there was an increase of more than three folds. The results indicate that a high level of NO acts mainly in a cGMP-independent way during the early phase (Additional file 6: Figure S4). Moreover, the importance of NO production is also reflected in its conserved increasing rate of production after injury of the very early stages such as stage 10 (gastrula), stage 20 (neurula) (Fig. 1g) and also during the late developmental stages such as stage 41 and later (Fig. 1e). High levels of NO production has potentially several other effects such as a nonspecific inflammatory response with antimicrobial activity or regulation of chemotaxis [71]. NO production then decreases after 15 min and returns to physiological state around 30 min. Interestingly at the same time, ROS production starts and continues [20, 72]. Wound healing is initiated with the rapid calcium release in the cells at the wound edge and the calcium wave spreads radially within seconds. The released calcium ions triggers the production of the actinomyosin ring that is responsible for the quick wound constriction [12]. The level of calcium returns to normal within a few minutes [73, 74]. The time sequence of calcium, NO and ROS production suggests a connection among these small molecules [12, 20, 72–74], but more thorough study are required to prove this hypothesis.

Interestingly, the same principle of calcium release followed by ROS has been observed in *Xenopus laevis* eggs after fertilization [75]. However, we have not seen NO production in stage 1 (egg) or at stage 5 (blastula) (Fig. 1g), following an injury to the embryo. We believe that NO may also be produced at these early stages, but the methods used to currently assess NO are limited in resolution and cannot detect its rapid and/or low concentration of release.

Temporal RNA-Seq analysis revealed that the expression of only a few genes were significantly changing (9% of 23,609 expressed genes) during the first 90 min after injury, with only 166 of them showing a maximum expression at 30 min after injury (Fig. 3h). Moreover, we identified the same set of genes also in the healing tissue after tail amputation at developmental stage 41 (Additional file 7: Figure S5). Interestingly, the list of genes overlapped nicely with the results obtained from another study [18]. The goal of the Ding et al. [18] study was to reveal dorsal and ventral specific transcriptomes at the gastrula stage, but because of long dissection time and parallel healing the authors revealed group of early injury response genes such as *jun*, *fos*, *egr1* and *junb*. Interestingly, expression of early response genes was also found in adult tissues 60 min post mortem [76], and as an artefact during dissociation of tissue [77, 78]. Based on the literature and our results, we speculate that these genes are generally important for cells dealing with stress conditions and therefore we propose that these genes should be referred to as “Stress response genes”.

### The middle phase of embryonic healing (30–90 min after injury)

During the early phase, the wound area is reduced to roughly 20% within the first 30 min after injury. In the middle phase, NO returns to the normal physiological level, where it has a potentially positive effect on the cells around the wound. RNA-Seq analysis identified a group of genes, whose expression started to increase 30 min after injury. These genes are responsible for cytokine production or defence response. Several genes such as *lep* and *igfbp2* were shown previously to be important for healing and/or regeneration [79–81] and are connected with cell metabolism. Leptin is a small 16 kDa peptide hormone, which acts via transmembrane receptors (Lep-R, also known as OB-R) and regulates an energy homeostasis [82]. Relations between NO and Lep have been previously studied. However, there is still no consensus on their interactions, as some of these studies have observed NO production by Lep stimulation, through PKA and MAPK activation [83], while others have shown an exact opposite effect – attenuation of NO production after Lep exposition [84, 85].

Comparison of the temporal expression profile of the control with those from NO inhibited embryos revealed three groups of affected genes (Fig. 4). The first group (Group 1′) contains genes such as *lep*, *mmp8* and *igfbp2* (mentioned above). The expression of these genes gradually increased during normal healing but was completely inhibited or delayed in NO inhibited embryos (Fig. 4b, Fig. 5j). This suggests the importance of NO as a gene expression regulator for cell metabolism and tissue remodelling. Our results showed that the *lep* gene serves as a downstream target of NO (Fig. 4h, i), which to our knowledge is the first time this has been observed. We performed *lep* loss-of-function experiments and results during wound healing were nearly identical with NO inhibition (Fig. 6e). Interestingly, the effect of *lep*-MO was extremely dependent on its concentration. Injection of a few nanograms below or above optimal concentration led to no phenotype or an inhibition of development at the neurula stage, which later led to death and cellular decay. We tested several options for combined NO – *lep* phenotype rescue using either NO donors or Lep protein/mRNA, but none of our experiments showed a significant improvement in healing capacity. We speculate, that the lack of an effect may be due to the requirement for a defined particular concentration of Lep to circumvent the effect of the given *lep*-MO concentration range.

The second group (Group 2′) is formed from Stress response genes. Embryos at stage 26 with inhibited NO production showed an increase in the expression of some of these genes in the whole embryos (e.g. *fos, jun*), more than 1.5-fold [86]. Dissected healing tissue showed at the beginning the same increase of gene expression at 30 min after injury as in control embryos. However, expression of these genes were turned off during the middle phase of healing in controls in contrast to the continuous growth of expression in NO inhibited embryos. We speculate that persistent cell stress response reflected by continuous expression of Stress response genes have an impact on wound closure and especially on the remodelling phase of wounded tissue. NO signal could be considered as back-signalling switch of cellular stress. The third group (Group 3′) consists of genes, which are downregulated in normal healing, but their level remains constant after NO inhibition. Unfortunately, biological interpretation of the roles of these genes during wound healing is much more complex and require further studies.

Subsequently, we wanted to elucidate the mechanism of NO on gene expression regulation during the middle phase of wound healing. NO has been shown to regulate gene expression through the cGMP-dependent pathway in many biological situations such as apoptosis [87], proliferation [88] and angiogenesis [89]. Based on our results, we concluded that this pathway is important during the middle phase of embryonic wound healing. We observed similar wound healing defects in embryos with chemically induced sGC inhibition or with *prkg1* loss-of-function phenotype. In addition, gene expression changes of selected candidates (*lep* and *fos*) showed identical profiles as embryos with inhibited NO production.

Morphological analyses using immunohistochemistry and collagen labelling revealed two different phenotypes in wound tissues. Control tissue showed proper cell sheet organization, basement membrane remodelling at the wound edges and complete restoration of laminin layer at the late phase. Chronical NO production inhibition caused abnormal basement membrane formation in embryos injected with MOs at the one cell stage. Even though the early phase of healing looks similar to control embryos, the middle and late phases revealed basement membrane formation defects, which resulted in delayed or missing wound closure. In the NO inhibited embryo, we speculate that the cells at the wound edge cannot move along the disrupted basement membrane, which is crucial for the fusion of the wound edge. Acute NO production inhibition achieved by TRIM inhibitor showed a different cellular behaviours at the wound edge. Cells at the wound edge formed “cell blobs” preventing cell movement during healing, which resulted in defects even during the early phase of healing. Laminin layer at the wound edge is intact, which supports cell movement defect. Replacement of collagen layer in the epidermis during middle phases of healing was found to be significantly reduced in NO inhibited embryos, which support previously discovered connection between NO and collagen synthesis [90].

An interesting phenomenon of adult wound healing is the migration and activity of immune cells. NO production is required for defence response/naïve immune activity during the early and middle phase of healing. Since embryos do not have a fully functional immune system, which would include a fully differentiated B and T cells that only develop at around 12 days post fertilization (stage 47) in *Xenopus laevis* [91], we hypothesize that NO acts differently in our experiments. Instead, during early development, primitive myeloid cells may provide a positive effect on defence and tissue remodelling. However, expression levels of gene markers of this cell type such as *mmp7*, *mmp9*, *spib* and *mpo* are not changed in NO inhibited embryos during the early and middle phases of embryonic healing. Surprisingly, their expression is changed after wound closure (more than 2 h after injury, Fig. 5i, j) and it suggests that there is a following late phase of wound healing which has not been sufficiently discussed in the available literature.

### The late phase of embryonic wound healing (more than 90 min)

The late phase of embryonic wound healing has not been defined yet. It starts after wound closure. The tissue beneath the wound site is remodelled and cells such as primitive myeloid cells migrate into the wounded place (Fig. 5). Two matrix metalloproteinases (gelatinases), *mmp7* and *mmp9* are used as markers for primitive myeloid cells [92–95]. Embryos with inhibited NO production showed a reduction of primitive myeloid cell migration to the wound site and suggests a reduction of Mmp7 and Mmp9 activity in remodelled wound tissue. In addition, RNA-Seq expression profiles of other *mmps* [96], which are important for tissue remodelling such as collagenases *mmp1* and *mmp8,* were changed at the late phase of healing in contrast to constant expression during early and middle phases. Expression of *mmp1* was previously shown to be regulated by laminin expression [97] and abnormal laminin production in NO inhibited embryos led to overexpression and potentially to detrimental over-activity of MMP1 enzyme in the wound tissue. Overexpression of *mmp8* was shown to cause chronic wounds [98], which is similar to its expression during the late phase of healing of NO inhibited embryos. All results together show not only existence of remodelling phase of embryonic wound healing but regulation of late phase by NO signalling during the early phase too.

### The relevance of NO healing properties

In this work, we described the role of NO during embryonic wound healing. We showed that NO is crucial for the early and middle healing phases and its activity depends on the quantity of its production. NO is not only required for proper wound healing. The other positive effect of NO during wound healing can also be achieved therapeutically through its supplementation with additional NO (for example using NO donors), which can significantly improve healing, especially in chronically non-healing tissues.

NO donor use has been found to improve wound healing in diabetic rats [99] and NOD-SCID (diabetic immunodeficient) mice [100]. Moreover, it was observed for *in vitro* wound healing assays that NO donors can speed up cell migration and collagen deposition [101, 102]. Application of sildenafil cream (inhibitor of cGMP specific phosphodiesterase type 5 - PDE5) on human epidermal injuries has been found to result in faster wound healing [103]. We were not able to reproduce a significant faster healing in embryos through the use of the NO donor, S-Nitroso-N-acetylpenicillamine (SNAP). We believe it is a matter of just finding the correct NO concentration and also the appropriate means of administration. However, owing to the fast rate of embryonic wound healing, it will be difficult to accurately measure the time difference for any increase in healing when using NO donors.

Perhaps the most attractive usage of NO donors is for human medicine. The relevance of NO donors for different human therapies was recently reviewed by Yang, et al. [104]. NO donors were applied, for example, during pressure ulcer treatment and the results showed statistically significant improvement of the speed of wound healing [105]. This makes NO donor an ideal potential treatment for patients with diabetic foot ulcers [106]. However, the current suggested mechanism of NO activity during wound healing has been only limited to mainly angiogenesis and inflammation. In this study, we also suggested here a new connection between upstream NO and downstream Lep. This could be relevant for understanding the molecular regulations during many chronical diseases such as high-blood pressure, diabetes and obesity in context with chronic wound healing problems. It appears that NO has more functions during healing, and therefore additional studies will be required if it is to be used in the future for routine targeted/personalized treatment.

## Conclusions

The main goal of this study was to identify the processes that are regulated by NO during the healing of embryos that lack a developed vascular and immune system. Our results showed that inhibition of NO led to later developmental defects and that NO is also essential for the regulation of other processes/systems other that the ones mentioned above. We observed that the production of NO during the early phase is crucial for the regulation of gene expression during the middle phase of healing. NO was also found to regulate the *de novo* expression of genes related to metabolism (such as *lep* and *igfbp2*). Additionally, inhibition of NO led to permanent stress of the cells around the wound edge, which is described by an increased expression of “Stress response genes” during the healing of the NO inhibited embryos. Additionally, we observed and characterized a new phase of healing, the late healing phase, which continues for hours after wound closure. The results provides new insight into the regulation of scarless embryonic healing.

## Methods

### Ethics statement

All animal experiments were performed in accordance with protocols approved by the animal committee of the Czech Academy of Sciences and were performed according to EU legislation (including animal handling guidelines and regulations).

### Embryo preparation

*Xenopus laevis* adults were obtained from the European Xenopus Resource Centre (EXRC) and grown in our breeding facility. *Xenopus laevis* embryos were obtained by *in vitro* fertilization [107]. Females were stimulated with 500 U of human chorionic gonadotropin (Sigma-Aldrich, CG10) and eggs were collected the following day. Eggs were *in vitro* fertilized using testes suspension and the jelly coats were removed by 2% cysteine treatment. After fertilization, the embryos were incubated in 0.1x MBS until gastrulation where they were incubated in 0.1x MBS medium with the addition of gentamicin (20 μg/mL, Sigma-Aldrich G1397). Embryos were incubated at 15 °C. Developmental stages were scored according to Nieuwkoop & Faber [108].

### NO staining

The middle region of five embryos were injured using a needle (outside diameter 0.45 mm, Gauge 26 × 1″). NO was stained with 5,6-Diaminofluorescein diacetate (DAF-2DA – Cayman 85,165). The stain was prepared as a 5 mM stock solution in DMSO, which was then diluted 1:150 in cultivation media. Embryos were transferred into media with DAF-2DA solution 15 min prior imaging. After that, embryos were fixed in 4% paraformaldehyde in phosphate buffer (PFA) and incubated for 30 min at the room temperature (e.g. time 0, embryos were transferred into DAF-2DA containing solution, cultivated for 15 min, and fixed immediately after injury). After fixation, embryos were washed two times for 5 min with phosphate buffer solution (PBS) with 1% Tween-100 buffer (PBT) and imaged using Leica MZ FLIII fluorescent stereoscope equipped with a Nikon digital sight DS-Fi2 camera. Fluorescent intensities around the wound site were analysed using FiJI (NIH, v1.52n) with Corrected Total Fluorescence (CTF) calculated using formula:

$$ \mathrm{CTF}=\mathrm{Integrated}\ \mathrm{Denisty}-\left(\mathrm{Area}\ \mathrm{around}\ \mathrm{the}\ \mathrm{wound}\ast \mathrm{Mean}\ \mathrm{fluorescence}\ \mathrm{of}\ \mathrm{background}\right) $$

The statistical significance was calculated relative to time 0 min using GraphPad Prism 7 and One-way ANOVA with Dunnett’s multiple comparisons test.

Similarly, three tadpoles at stage 41 were anaesthetized for 5 min using 0.025% MS222 (Tricaine, Sigma-Aldrich, E10521) diluted in 0.1x MMR medium and about 50% of the tail length from the tip was amputated using a scalpel. Tadpoles were then transferred to 0.1x MMR with gentamicin (50 μg/ml) containing NO staining solution. The site of amputation of tails was imaged at 15, 30, 60, 180 min post amputation.

### Morpholino injection

All morpholino oligonucleotides (MOs) were purchased from Gene Tools, LLC (Phliomath, OR, USA) and diluted to a final concentration of 17 ng/μl. The amount of injected MO was: 2 nl of standard control MO (5′-CCTCTTACCTCAGTTACAATTTATA-3‘ designed by Gene Tools), 2 nl of *nos3*-MO (5’-AAAAGCCAAGCACTACTCACCGTTT-3′ [86]), 1 nl of *nos1*-MO (5′-TGGCTAAAAGAACACAGGACATCAA-3′ [109]), 2 nl of *prkg1*-MO (5′-TTCAGCTTCAATGCTCATACCTGCC-3′ [86]), 0.3 nl of *lep*-MO (5′-TTGCAGTGTCCATGTTTCTCACCTG-3′) and 3 nl of mixture *nos1* + *nos3*-MO in 1:2 ratio. Injections were performed at the one-cell stage embryos for all cases. The effectivity of *nos1*-MO and *nos3*-MO were tested in previous studies [86, 109]. The specificity of *prkg1*-MO and *lep*-MO were tested using PCR (Additional file 12: Figure S8).

### Chemical inhibition of NO production and sGC activity

Chemical inhibitors were selected to block NO production and sGC activation.

1-(2-Trifluoromethylphenyl) imidazole (TRIM, Sigma-Aldrich, T7313) is a reversible inhibitor that preferentially blocks NOS2 and NOS1 activity and also partially blocks NOS3 activity. TRIM was prepared as a 1 M stock solution in dimethyl sulfoxide (DMSO) and its final concentration in media was 2 mM. 1H -[1, 2, 4] Oxadiazolo [4,3-a]quinoxalin-1-one (ODQ, Sigma-Aldrich, O3636) is an irreversible and competitive inhibitor of soluble guanylate cyclase (sGC). ODQ was prepared as 100 mM stock solution in DMSO and used at a final concentration of 0.2 mM.

The vitelline membranes were manually removed from embryos at stage 24 using forceps (FST, 11203–23). Embryos were incubated in 0.1x MBS media with added inhibitors 1 h prior to wounding and during the whole time of monitoring of healing. Media were changed immediately after wounding and again at 30, 90 and 180 min pw to avoid inhibition of wound healing caused by decaying material. The effectivity of TRIM to inhibit the production of NO was tested by staining for NO using DAF-2DA (Additional file 13: Figure S9).

### Wound healing monitoring

Wounds were created using a needle (outside diameter 0.45 mm, Gauge 26 × 1″) puncture to obtain constant damage size in the in the middle of the lateral side of the embryo. Embryos at stage 26 were injured and wound closing was documented using brightfield imaging on stereomicroscope (Nikon SMZ 1500) with Nikon digital sight DS-Fi1 camera. Size of the wound closure was measured in pixels using FiJI (NIH) software and normalized relative to 0 min pw (immediately after injury). Each wound size was measured three times, with the values plotted on graphs representing an average from at least three embryos. Comparison of two linear mixed models was done using the R package lme4 (v1.1–21) [110] for finding the difference between conditions. Condition and time were used as fixed variables while sample type was used as a random variable.

### Gene expression analysis of healing tissue

RNA-Seq experiment was designed as differential analysis of temporal expression profiles between control embryos and embryos with inhibited NO production (either using *nos1* + *nos3*-MO or TRIM treatment). Embryos at stage 26 were scratched by forceps (at the middle ventral side). The tissue surrounding the wound was manually dissected, and dissected tissues from five different embryos were pooled as one biological replicate.

### RNA isolation

Dissected healing tissues (red rectangle on Fig. 3a and Fig. 4a) were collected into 2 ml tubes containing pre-cooled beads (Qiagen Stainless Steel Beads, 5 mm, 69,989) and stored at − 80 °C freezer. Samples were homogenized using TissueLyser LT (Qiagen) for 5 min at 50 Hz. Total RNA was isolated using 1 ml of TRI Reagent (Sigma-Aldrich, T9424) following the manufacturer’s manual. The RNA pellet was then dissolved in 40 μl DNAse solution (32 μl Nuclease-free water, 4 μl 10x reaction buffer, 4 μl DNAse I, Sigma-Aldrich, AMPD I), and incubated at 37 °C for 30 min. Afterwards, 40 μl of 8 M LiCl (Sigma-Aldrich, L7026) were added for RNA precipitation. This solution was incubated overnight in − 20 °C freezer and then centrifuged for 30 min at 16,000 g. The supernatant was removed, and the RNA was then washed twice with 1 ml of 80% ethanol followed by centrifugation for 30 min. The final total RNA was then diluted in 20 μl of 1xTE buffer (Invitrogen, 12,090–015). The concentration of RNA was measured using Nanodrop 2000 (Thermo Scientific), and the quality of RNA was analysed using Fragment Analyzer (AATI, Standard Sensitivity RNA analysis kit, DNF-471).

### RNA-Seq

#### Library prep

Wounded tissue samples were collected at 0, 30, 60 and 90 min pw from control and NO inhibited embryos and their RNA was isolated according to the protocol mentioned above. RNA-Seq experiment was performed as two separated experiments. In the first experiment, only control samples in biological triplicate were analysed. Libraries were prepared from 200 ng of total RNA using SureSelect Strand-Specific RNA Library Prep for Illumina Multiplexed Sequencing (Agilent, G9691) according to manufacture protocol, which utilized poly-A selection of RNA. Final libraries were equimolary pooled and sequenced on NextSeq 500 using 2x75bp HighOutput mode. The data are available at NCBI’s Gene Expression Omnibus [111] under GEO Series accession number GSE116667 (https://www.ncbi.nlm.nih.gov/geo/query/acc.cgi?acc=GSE116667). The second experiment was performed similarly, where samples from both control and NO inhibited embryos were prepared in biological duplicates. A total of 500 ng of total RNA was used for poly-A selection (NEB #E7490S). The libraries were then prepared using NEBNext® Ultra™ Directional RNA Library Prep Kit for Illumina® (NEB #E7420S) according to manufacture protocol. Again, final libraries were equimolary pooled and sequenced on NextSeq 500 using 2x75bp HighOutput mode and the final data has been deposited available under GEO Series accession number GSE116678 (https://www.ncbi.nlm.nih.gov/geo/query/acc.cgi?acc=GSE116678).

The gene expression during tail regeneration was studied too. Tissue from the amputated tail of the stage 41 tadpoles were collected at 0, 0.5, 1, 1.5, 3, 6, 24, 72 and 168 h post amputation. RNA was then isolated and libraries prepared for RNA-Seq as similarly described for the wound healing experiment (Additional file 7: Figure S5).

#### Data analysis

On average, approximately 20 M reads per sample were obtained after filtering out for low quality reads and adaptor sequence removal, using TrimmomaticPE (v. 0.36) [112] with the parameters “CROP:70 HEADCROP:15 ILLUMINACLIP:~/TruSeq-PE3.fa:2:30:10 LEADING:3 TRAILING:3 SLIDINGWINDOW:4:15 MINLEN:36”. Ribosomal RNA reads were filtered out using Sortmerna (v. 2.1b) [113] (default parameters) and the cleaned reads were then aligned using STAR (v. 2.5.2b) [114] to the *Xenopus laevis* genome version 9.1 and annotation version 1.8.3.2 (http://www.xenbase.org/, RRID:SCR_003280) [115]. A count table was then generated using the python script htseq-count (v. 0.6.1p1) [116] with the parameter “–m union”. Differentially expressed genes (DEGs) were analysed by DESeq2 (v. 1.15.51) [117] using the design formula parameter “ ~ time”, with time defined as a factor. DEGs were identified using default function DESeq, with parameters “test = ‘LRT’, reduced = ~ 1, fitType = ‘local’“. An expressed gene was defined as one that had a mean normalized counts of at least five, while DEGs were defined as a genes with padj < 0.1. Similarly, to identify genes which are differentially expressed between two conditions (control vs. TRIM, control vs. *nos1 + nos3*-MO), design formula was set to “ ~ condition + time + time:condition” and parameters for DESeq function were set to “test = ‘LRT’, reduced = ~ condition, fitType = ‘local’“. The final list of genes was controlled manually during the clustering process.

#### Clustering

The optCluster (v1.1.1, R v3.4.2) package [118], using the Diana clustering method and ten requested clusters, was used to group the expression profiles of the DEGs identified from the first control experiment. Clustering was performed using the relative proportion of the averaged normalized counts across the time points. The produced ten clusters (Additional file 8: Figure S6) were then manually analysed and clusters that showed similar profiles were merged together to produce the final cluster profile (Additional file 1: File S1).

The Regularized log transformation of the normalized counts of the DEGs identified from the second experiment were clustered using degPatterns from the R package DEGreport (v1.13.8) [119]. The function was run using the default parameters, except that the produced clusters were allowed to contain a minimum of one gene representative (minc = 1), similar clusters were merged based on a correlation of expression of 0.7 (cutoff = 0.7) and outliers within the clusters were removed (reduce = TRUE). The produced clusters (*n* = 36) (Additional file 9: Figure S7) were then manually analysed and clusters that showed similar profiles were merged together to produce the final cluster profiles (Additional file 3: File S2).

#### Gene ontology analysis

Gene Ontology (GO) Enrichment analysis was performed on the gene members from each unique cluster using the Gorilla webserver [120, 121]. The biological processes were assessed using the human database as a reference, an unranked list of the human orthologues of the *Xenopus laevis* cluster members, and a background set containing the human orthologues for all of the *Xenopus laevis* genes (Additional file 10: File S3). Results were filtered to only include *p*-values less than 1e-3. The Revigo webserver [122] was then used to summarize the significant p-value ranked GO terms as a treemap. Gene Ontology terms were collapsed if they shared 0.7 similarity while using the SimRel method to assess for similarity. The whole of the UniProt database (2017) was utilized to analyse for the size of the GO terms.

### RT-qPCR

The RT-qPCR analysis was performed using 50 ng of total RNA obtained from dissected healing tissues (red rectangle on Fig. 3a and Fig. 4a) prepared from an independent experiment (another fertilization/different female than RNA-Seq) using three biological replicates. RNA Spike I (0.5 μl, TATAA Biocenter) was added before reverse transcription to test inhibition in enzymatic reactions. Reverse transcription was performed using SuperScript III (Invitrogen) according to the manufacturer’s protocol in 10 μl volume. Synthesized cDNA was diluted 10 times in 1xTE buffer and 2 μl of final cDNA was added to the qPCR reaction (2x SYBRGreen mix, TATAA Biocenter, 400 nM primers mix and Nuclease-free water to final volume 6 μl). Primer sequences are listed in the Additional file 11: Table S1. Protocol for qPCR was: 1 min at 95 °C; 50 cycles of 95 °C for 3 s, 60 °C for 30 s and 72 °C for 10 s; followed by melting curve analysis.

### In situ hybridization

Whole-mount *in situ* hybridization was performed according to Sive et al. [123] protocol. List of clones which were used for analysis, their source, restriction enzyme used for linearization and RNA polymerases used for generation of RNA in sense or antisense strand can be found in Table 1. Eight healing embryos per condition were fixed in PFA overnight, dehydrated into methanol and stored at − 20 °C freezer. The proteinase K treatment was not used. Samples were imaged on stereomicroscope (Nikon SMZ 1500) and processed using Zoner Photo Studio 17. In all cases, sense probes showed the absence of staining. Blue signal around the wound site were analysed using FiJI (NIH, v1.52n). To measure the signal, the image colour was first inverted and the intensity of the signal was then calculated using the average of the values from three independent measurements using the formula:

$$ \mathrm{Intensity}\ \mathrm{of}\ \mathrm{signal}=\mathrm{Integrated}\ \mathrm{Density}\ \mathrm{around}\ \mathrm{wound}-\mathrm{Integrated}\ \mathrm{Density}\ \mathrm{inside}\ \mathrm{wound}-\left[\left(\mathrm{Area}\ \mathrm{around}\ \mathrm{wound}-\mathrm{Area}\ \mathrm{inside}\ \mathrm{wound}\right)\ast \mathrm{Mean}\ \mathrm{fluorescence}\ \mathrm{of}\ \mathrm{background}\right] $$

**Table 1:** List of constructs and enzymes used for *in situ* hybridization probe preparation

Gene	Source	Antisense probe	Sense probe
*fos. S*	Dharmacon #MXL1736–202773077	SalI, T7	NotI, SP6
*mmp7.L*	Dharmacon #MXL1736–202774696	KpnI, T7	XhoI, SP6
*mmp9.S*	EXRC Number 851 XB-CLONE-2041688	XmaI, T7	XhoI, SP6

The statistical difference was calculated relative to the control using GraphPad Prism 7 and One-way ANOVA with Dunnett’s multiple comparisons test.

### Immunohistochemistry

Five healing embryos per condition were fixed in PFA overnight, washed three times in PBT and mounted into 4% agarose diluted in water. Next, 150 μm sections were prepared using Leica Vibratome (VT1000 S), and immunohistochemistry was performed according to Sive et al. [123] protocol using primary antibodies against β-catenin (1:1000, Sigma-Aldrich T9026), laminin (1:150, Sigma-Aldrich L9393) and actin - Alexa Fluor 488 phalloidin (1:1000, Life Technologies A12379). The secondary antibody Alexa 488 goat anti-rabbit (1:500, Life technologies A11008) was used for laminin and β-catenin staining. Samples were imaged using Carl Zeiss LSM 880 NLO microscope and images were processed using Zen (Zeiss) and Zoner Photo Studio 17 software.

### Collagens analysis

Six embryos per condition at stage 26 were injured and fixed after 60 min in 4% PFA overnight. Samples were washed three times in PBS, dehydrated in 70% ethanol and embedded in paraffin. 50 μm histological sections were then prepared using Leica Microtome (RM 2255) and mounted on microscope slides. Sections were deparaffined and stained using Masson’s trichrome staining kit (Sigma-Aldrich, HT15). Slides were washed in demineralized water and incubated for 5 min in fuchsin solution. Slides were repeatedly washed in demineralized water and incubated in phosphowolfram/phosphomolybdene acid solution for 5 min followed by aniline blue solution for 5 minutes. Slides were then washed for 2 minutes in 1% acetic acid and dehydrated in 100% ethanol. Dehydrated samples were washed two times in 100% xylene and covered with DPX solution (Sigma-Aldrich, 44,851). Slides were imaged using Carl Zeiss AxioZoom V16 Microscope and the images processed using Zen (Zeiss) and FiJI (NIH, v1.52n) softwares. The coverage of collagen within the wound was calculated as the length of the dark collagen layer divided by the total length of the wound. It was measured using the average from at least five slices from each embryo. The statistical significance was calculated relative to the control using GraphPad Prism 7 One-way ANOVA with Dunnett’s multiple comparisons test.

## Supplementary information

**Additional file 1.** File S1 Information about various expression profiles for all genes and a complete list of enriched GO terms in Group 1 – 6

**Additional file 2.** Figure S1 REViGO analysis of enriched GO terms. Gorilla was used to determine enrichment of gene ontology terms, followed by summarization using REViGO. (A) Group 1 (B) Group 2 (C) Group 3 (D) Group 4 (E) Group 5. Similar GO terms between and within groups have the same colors

**Additional file 3.** File S2 Information about various expression profiles for all genes and a complete list of enriched GO terms in Group 1’-3’

**Additional file 4:.** Figure S2 REViGO analysis of enriched GO terms. Gorilla was used to determine enrichment of gene ontology terms, followed by summarization using REViGO. (A) Group 1’ (B) Group 2’ (C) Group 3’. Similar GO terms between and within groups have the same colors

**Additional file 5.** Figure S3 Actin staining of injury in embryos with inhibited NO production. (A) Acute NO inhibition using TRIM causes abnormal morphology of cells at the wound edge (white circle). (B) Chronic NO inhibition using MOs caused overproduction of actin around the wound edge and formation of abnormal structures inside the injury (marked by white arrows) (Scale bars = 100 μm)

**Additional file 6. **Figure S4 Monitoring of wound closing and changes in gene expression after inhibition of NO pathway. (A) Scheme of NO pathway with labelled inhibitors/gene specific MO which were used in experiments. (B) Control embryos, embryos with inhibited sGC using ODQ 1 hour before injury and embryos injected with *prkg1*-MO were injured using a needle at stage 26. (C) Wound closing was documented using brightfield imaging on stereomicroscope. (D) Relative wound closure was calculated as the ratio between the size of the wound in 0 minute pw (E) and 30 minutes pw (at least six replicates per condition, mean with standard deviation, the statistical difference between the groups is derived from two linear mixed models). (F) RT-qPCR comparison of temporal expression profiles of *fos* and *lep* (data are normalized to 0 minutes pw in controls, three replicates, geometric mean with geometric standard deviation, two-sided t-test from log2 values of relative expression between inhibited samples and control in 120 minutes pw). ****- *p* < .0001, * - *p* < .05, n.s. - *p* > .05. pw – post wounding

**Additional file 7. **Figure S5 Expression of genes from the Group 4 during regeneration. Expression of genes from the Group 4 (Fig. 3h) were analyzed during regeneration of amputated tail at stage 41 using RNA-Seq. The same representative genes (*fos* - green, *jun* - violet, *egr1* - blue) are shown

**Additional file 8.** Figure S6 Complete cluster profiles of the temporal gene expression from the control embryos. Clusters were produced using the optcluster function on the relative proportion of the averaged counts across the time points. The plotted y-axis however represents the z-score of the regularized log transformation of the normalized counts

**Additional file 9.** Figure S7 Complete cluster profiles of the temporal gene expression from the control and NO inhibited embryos. Clusters were produced using the degPatterns function on the regularized log transformation of the normalized counts

**Additional file 10.** File S3 List of genes used as a background during GO term enrichment analysis

**Additional file 11.** Table S1 List of primers used for RT-qPCR

**Additional file 12. **Figure S8 Test of specificity of *prkg1*-MO and *lep*-MO. (A, B) Function of MOs were analysed using RT-PCR and PCR products were visualized using gel electrophoresis. (A) Picture showed shorter product after usage of *prkg1*-MO. (B) Picture showed clearly that intron stayed unspliced after usage of *lep*-MO

**Additional file 13. **Figure S9 Test of the effectivity of TRIM to inhibit the production of NO and for the specificity of DAF-2DA to NO. (A) Control embryos at stage 26, embryos with inhibited production of NO using TRIM (6 mM) 1 hour before injury were injured using a needle, incubated in media with DAF-2DA solution for 10 minutes, fixed and imaged. (B) The intensity of signal were analysed (t-test). (C) Similarly, tails of tadpoles at stage 41, tadpoles with inhibited production of NO using TRIM (1 mM) or tadpoles with inihibited sGC with ODQ (100 μM), were amputated and incubated in media with DAF-2DA solution for 15 minutes, fixed and imaged. (D) The intensity of signal were analysed (t-test). **** - *p* < .0001, ** - *p* < .01, * - *p* < .05

## Data Availability

The transcriptome sequencing data from this study are available at NCBI’s Gene Expression Omnibus [111] under GEO Series accession number GSE116667 (https://www.ncbi.nlm.nih.gov/geo/query/acc.cgi?acc=GSE116667) and GSE116678 (https://www.ncbi.nlm.nih.gov/geo/query/acc.cgi?acc=GSE116678).

## Abbreviations

cGMP: Cyclic guanosine monophosphate
DAF-2DA: 5,6-Diaminofluorescein diacetate
DEGs: Differentially expressed genes
GO: Gene ontology
MMPs: Matrix metalloproteinases
MO: Morpholino oligonucleotide
NO: Nitric oxide
NOS: Nitrix oxide synthase
ODQ: 1H-[1,2,4] Oxadiazolo [4,3-a]quinoxalin-1-one
pa: Post amputation
PRKG1: Protein kinase cGMP-dependent 1
pw: Post wounding
ROS: Reactive oxygen species
sGC: Soluble guanylate cyclase
TRIM: 1-(2-Trifluoromethylphenyl) imidazole
