# Supplementary figures and images for: The role of nitric oxide during embryonic wound healing
Source: BMC Genomics. 2019 Nov 6;20:815. doi: 10.1186/s12864-019-6147-6 (PMC6836512; doi:10.1186/s12864-019-6147-6)

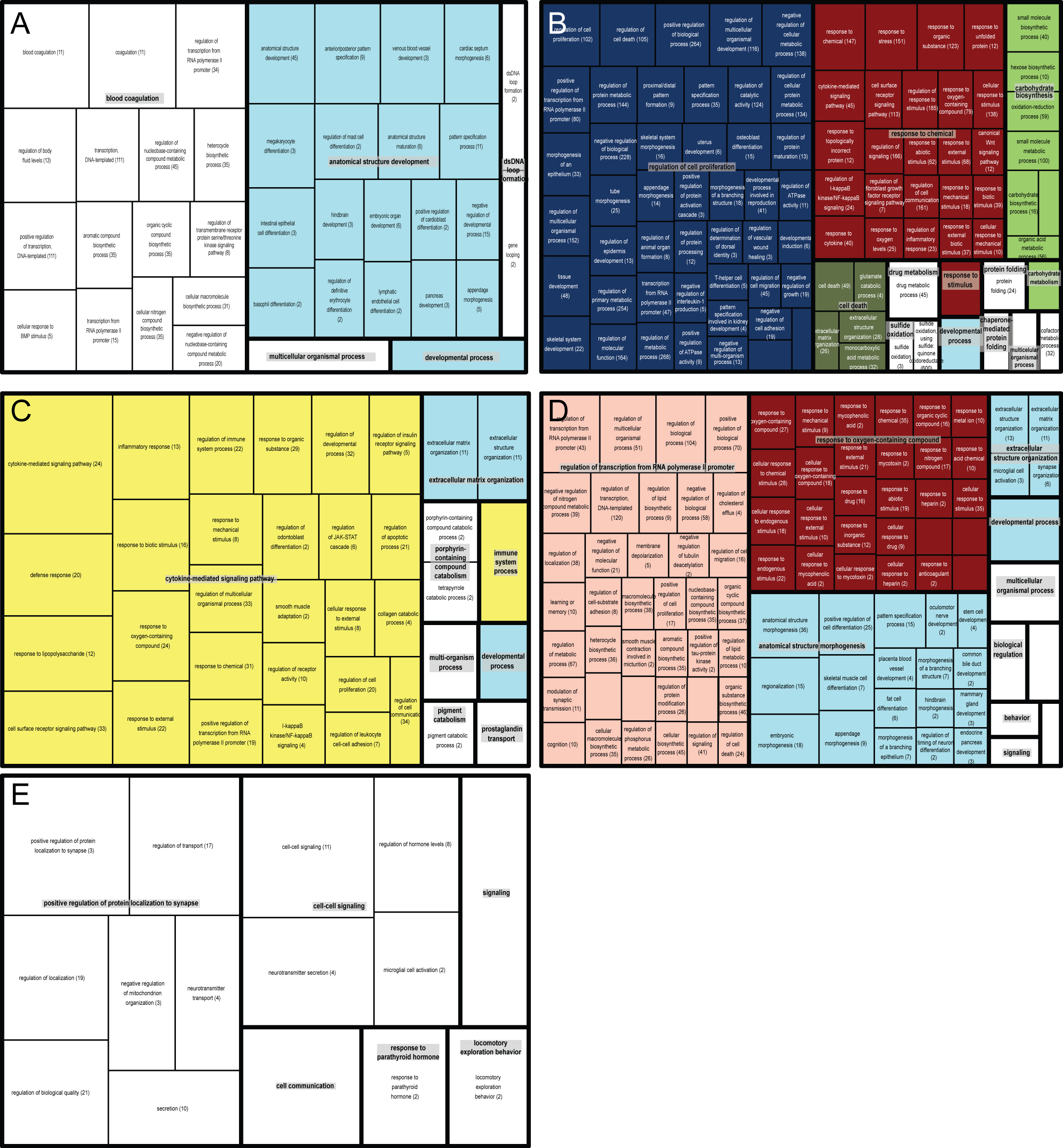

Supplement: Supplementary file 2 — Additional file 2. Figure S1 REViGO analysis of enriched GO terms. Gorilla was used to determine enrichment of gene ontology terms, followed by summarization using REViGO. (A) Group 1 (B) Group 2 (C) Group 3 (D) Group 4 (E) Group 5. Similar GO terms between and within groups have the same colors [file 12864_2019_6147_MOESM2_ESM.png]

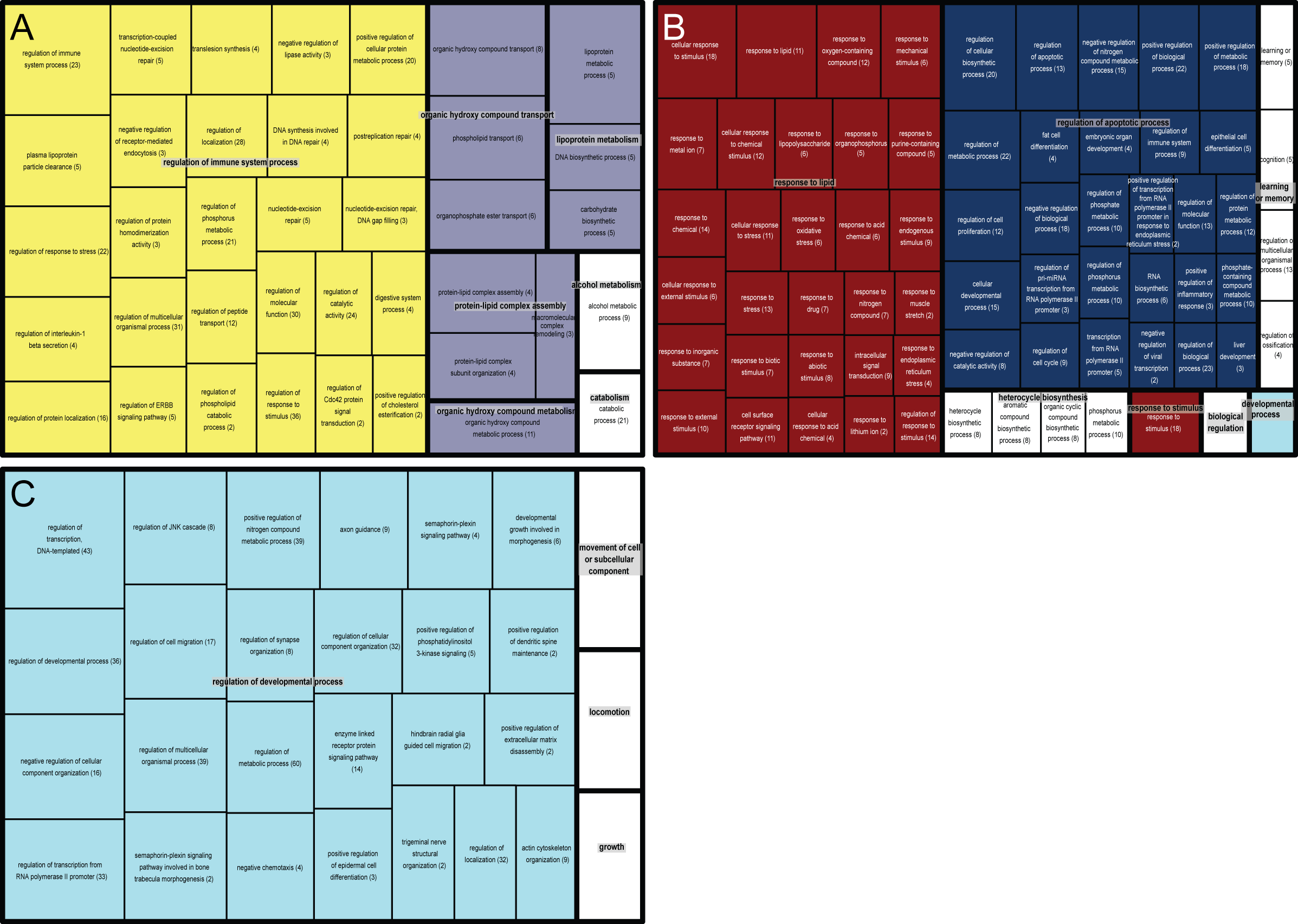

Supplement: Supplementary file 4 — Additional file 4:. Figure S2 REViGO analysis of enriched GO terms. Gorilla was used to determine enrichment of gene ontology terms, followed by summarization using REViGO. (A) Group 1’ (B) Group 2’ (C) Group 3’. Similar GO terms between and within groups have the same colors [file 12864_2019_6147_MOESM4_ESM.png]

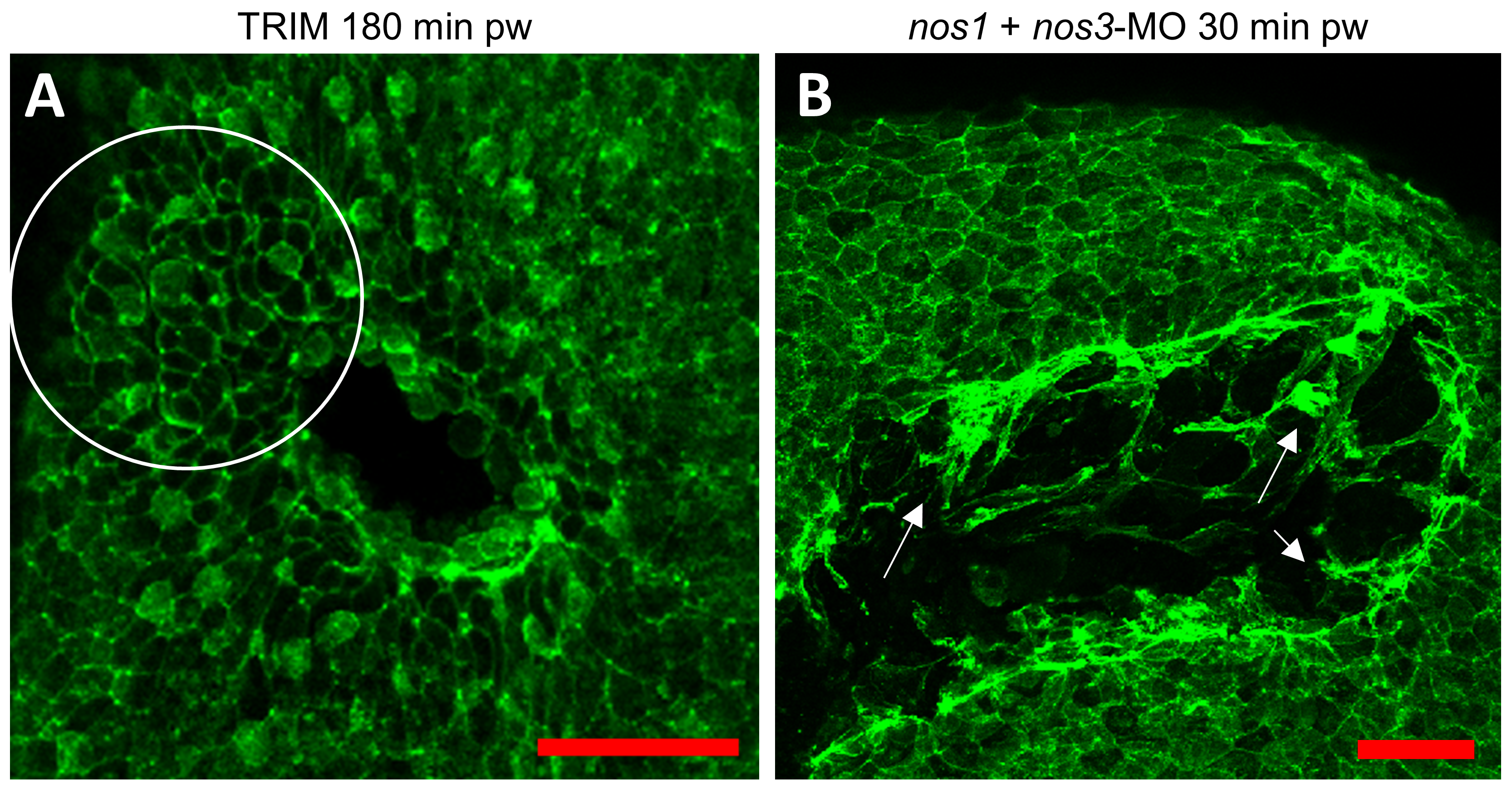

Supplement: Supplementary file 5 — Additional file 5. Figure S3 Actin staining of injury in embryos with inhibited NO production. (A) Acute NO inhibition using TRIM causes abnormal morphology of cells at the wound edge (white circle). (B) Chronic NO inhibition using MOs caused overproduction of actin around the wound edge and formation of abnormal structures inside the injury (marked by white arrows) (Scale bars = 100 μm) [file 12864_2019_6147_MOESM5_ESM.png]

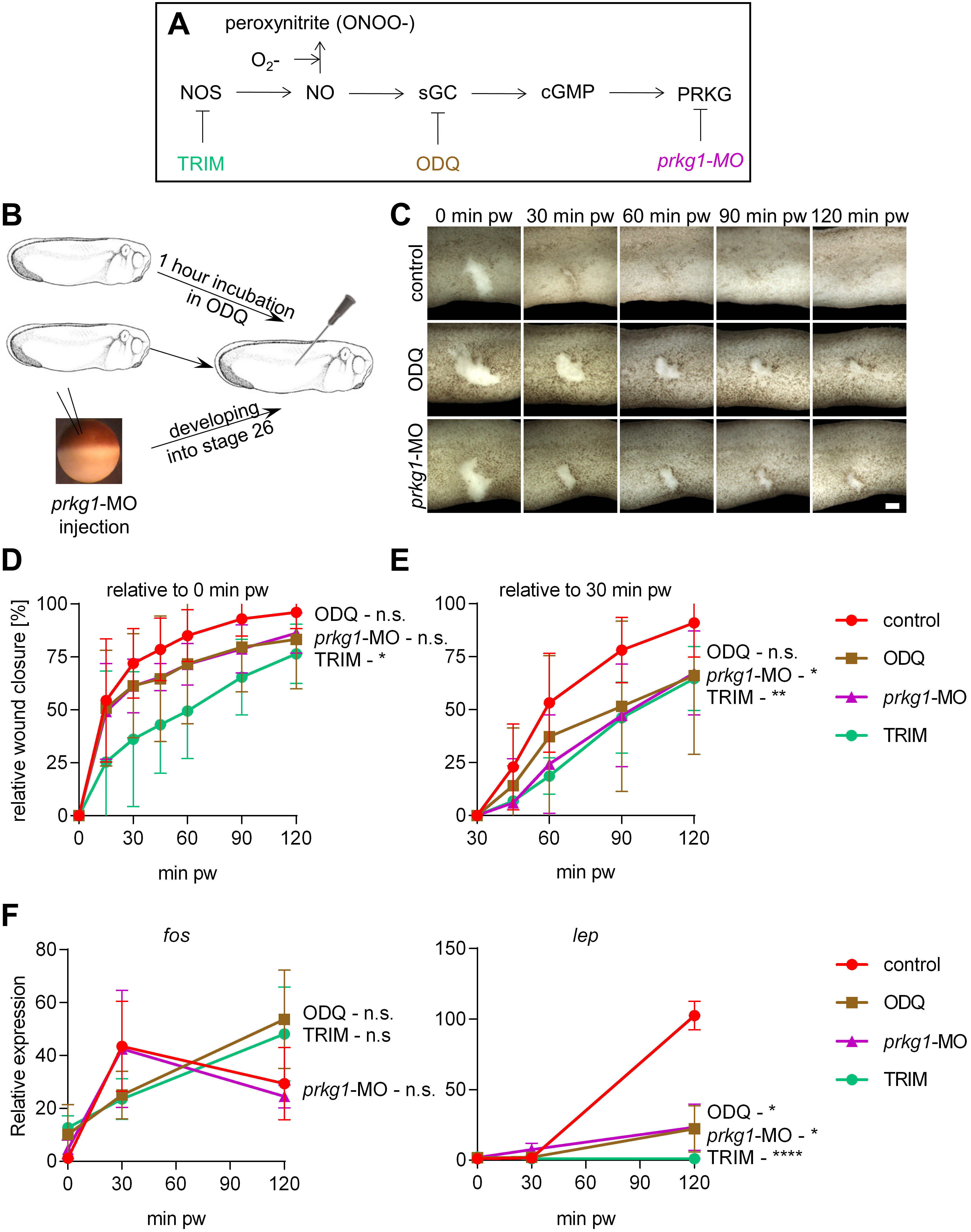

Supplement: Supplementary file 6 — Additional file 6. Figure S4 Monitoring of wound closing and changes in gene expression after inhibition of NO pathway. (A) Scheme of NO pathway with labelled inhibitors/gene specific MO which were used in experiments. (B) Control embryos, embryos with inhibited sGC using ODQ 1 hour before injury and embryos injected with prkg1-MO were injured using a needle at stage 26. (C) Wound closing was documented using brightfield imaging on stereomicroscope. (D) Relative wound closure was calculated as the ratio between the size of the wound in 0 minute pw (E) and 30 minutes pw (at least six replicates per condition, mean with standard deviation, the statistical difference between the groups is derived from two linear mixed models). (F) RT-qPCR comparison of temporal expression profiles of fos and lep (data are normalized to 0 minutes pw in controls, three replicates, geometric mean with geometric standard deviation, two-sided t-test from log2 values of relative expression between inhibited samples and control in 120 minutes pw). ****- p < .0001, * - p < .05, n.s. - p > .05. pw – post wounding [file 12864_2019_6147_MOESM6_ESM.png]

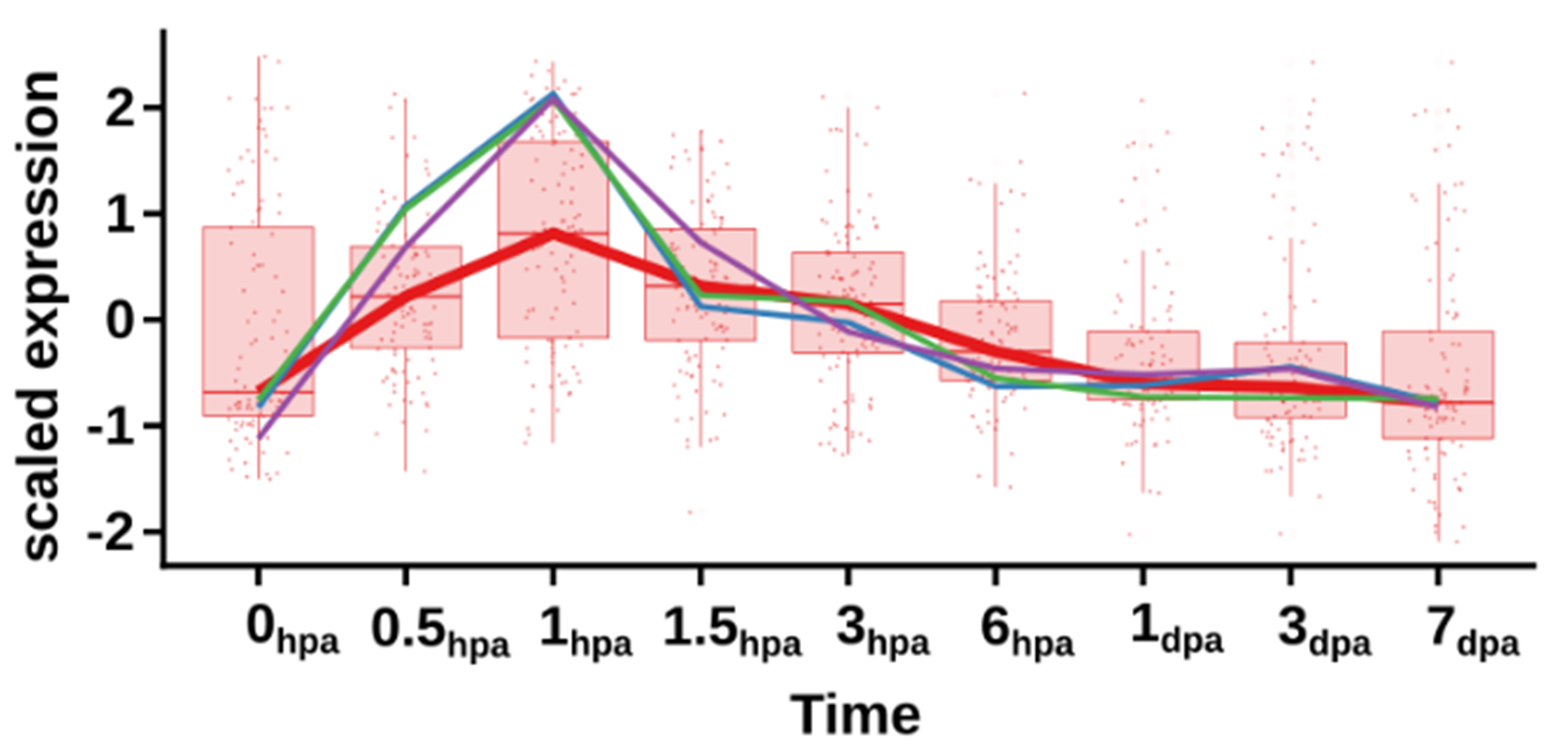

Supplement: Supplementary file 7 — Additional file 7. Figure S5 Expression of genes from the Group 4 during regeneration. Expression of genes from the Group 4 (Fig. 3h) were analyzed during regeneration of amputated tail at stage 41 using RNA-Seq. The same representative genes (fos - green, jun - violet, egr1 - blue) are shown [file 12864_2019_6147_MOESM7_ESM.png]

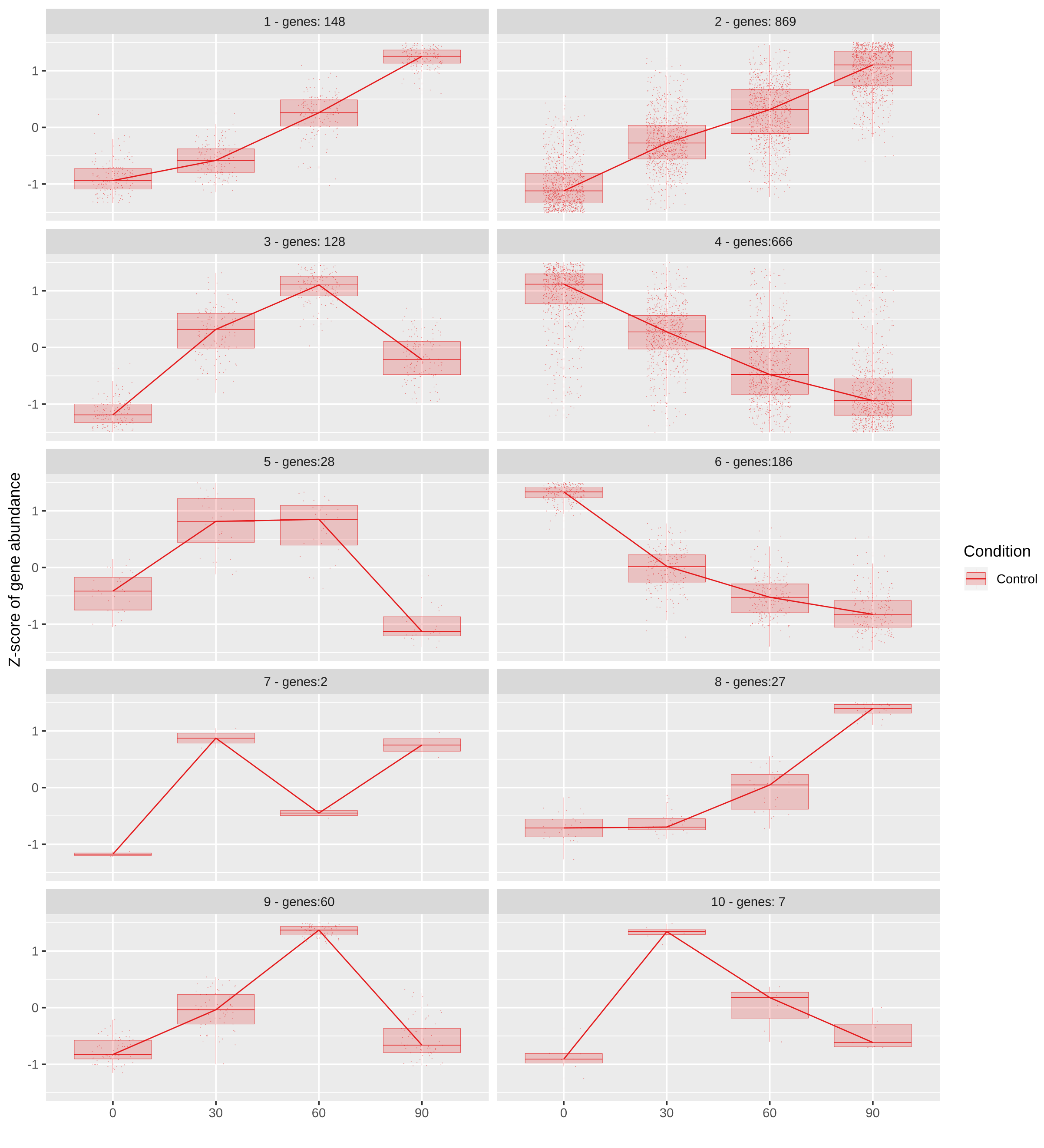

Supplement: Supplementary file 8 — Additional file 8. Figure S6 Complete cluster profiles of the temporal gene expression from the control embryos. Clusters were produced using the optcluster function on the relative proportion of the averaged counts across the time points. The plotted y-axis however represents the z-score of the regularized log transformation of the normalized counts [file 12864_2019_6147_MOESM8_ESM.png]

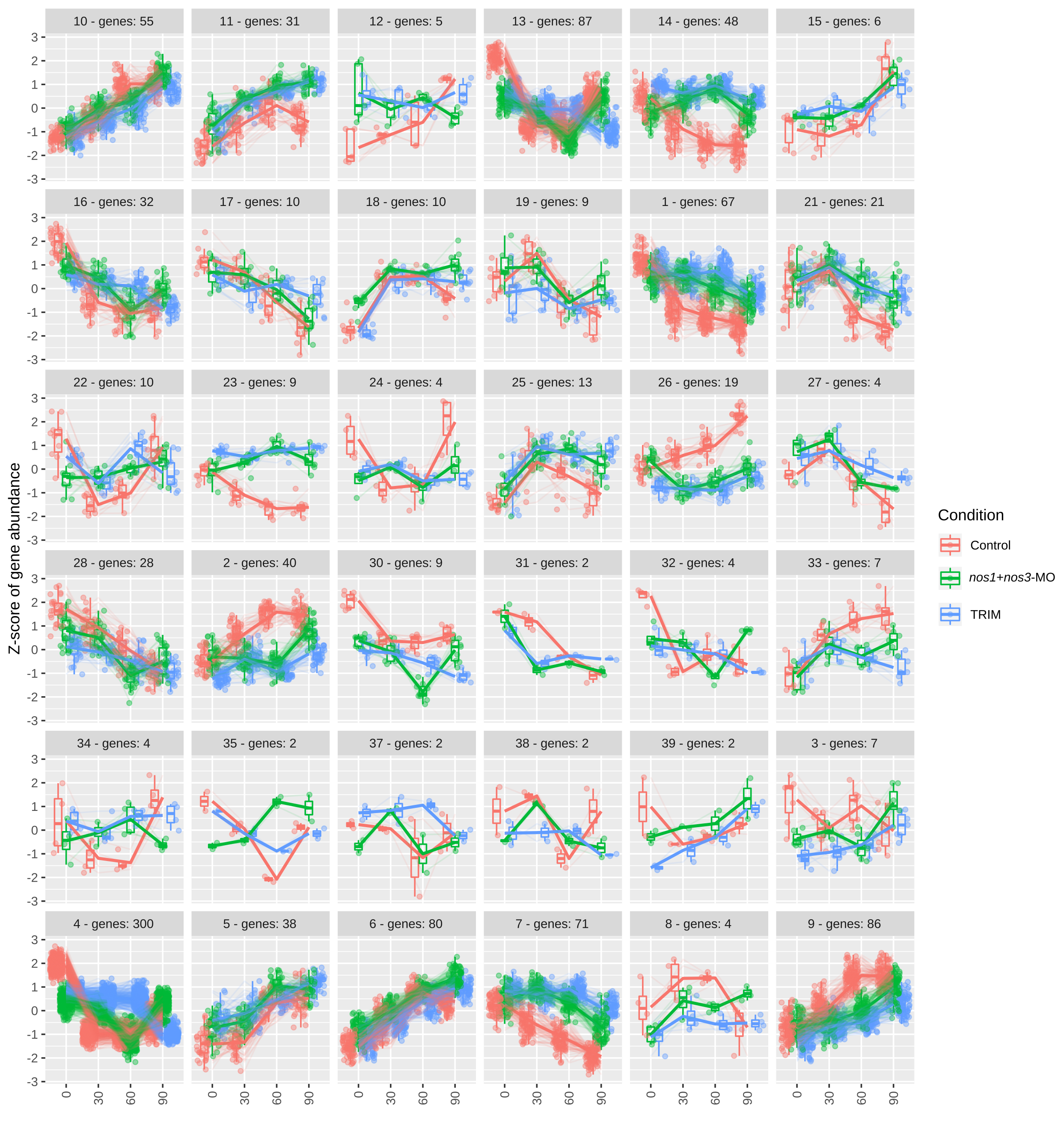

Supplement: Supplementary file 9 — Additional file 9. Figure S7 Complete cluster profiles of the temporal gene expression from the control and NO inhibited embryos. Clusters were produced using the degPatterns function on the regularized log transformation of the normalized counts [file 12864_2019_6147_MOESM9_ESM.png]

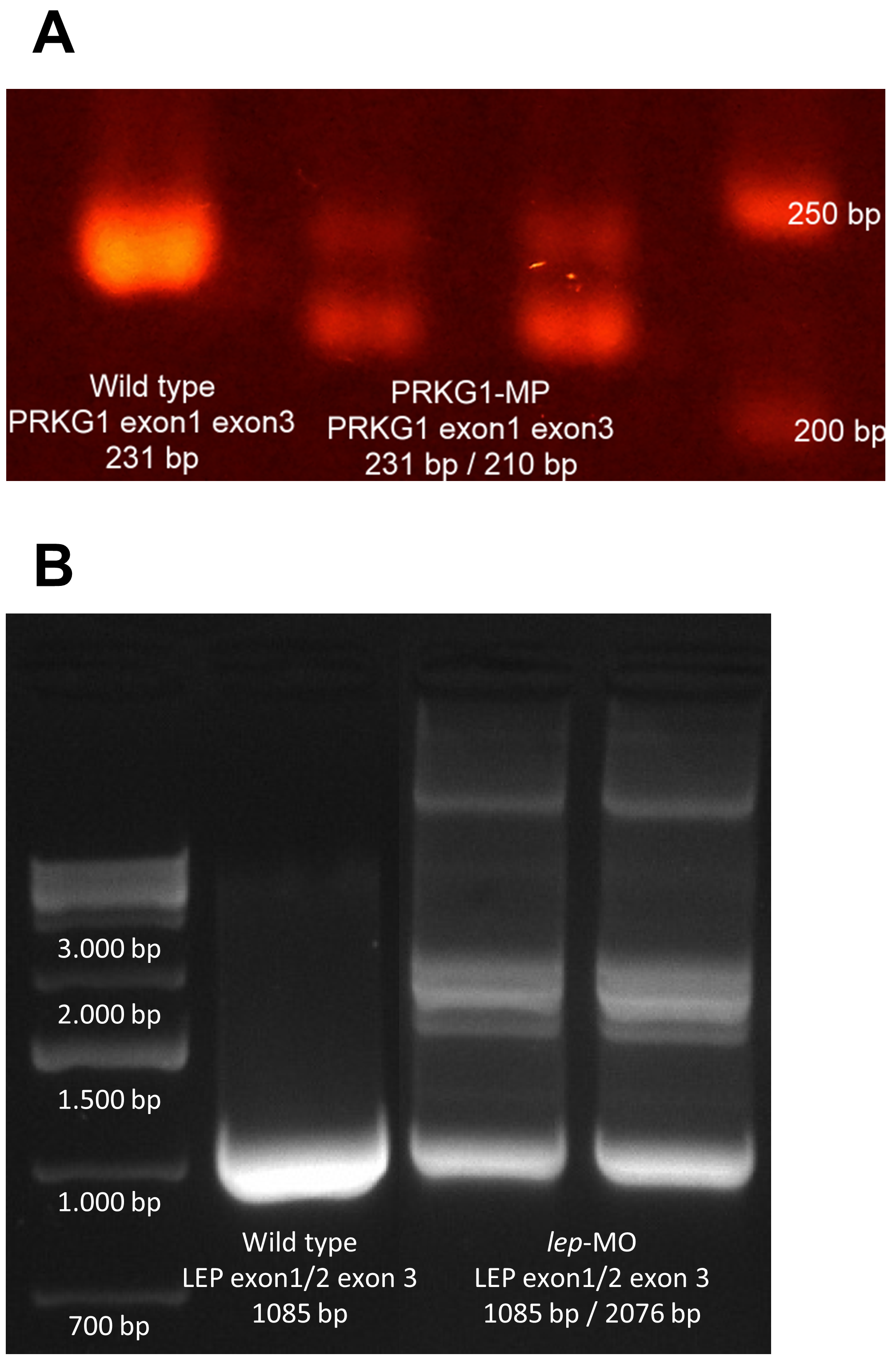

Supplement: Supplementary file 12 — Additional file 12. Figure S8 Test of specificity of prkg1-MO and lep-MO. (A, B) Function of MOs were analysed using RT-PCR and PCR products were visualized using gel electrophoresis. (A) Picture showed shorter product after usage of prkg1-MO. (B) Picture showed clearly that intron stayed unspliced after usage of lep-MO [file 12864_2019_6147_MOESM12_ESM.png]

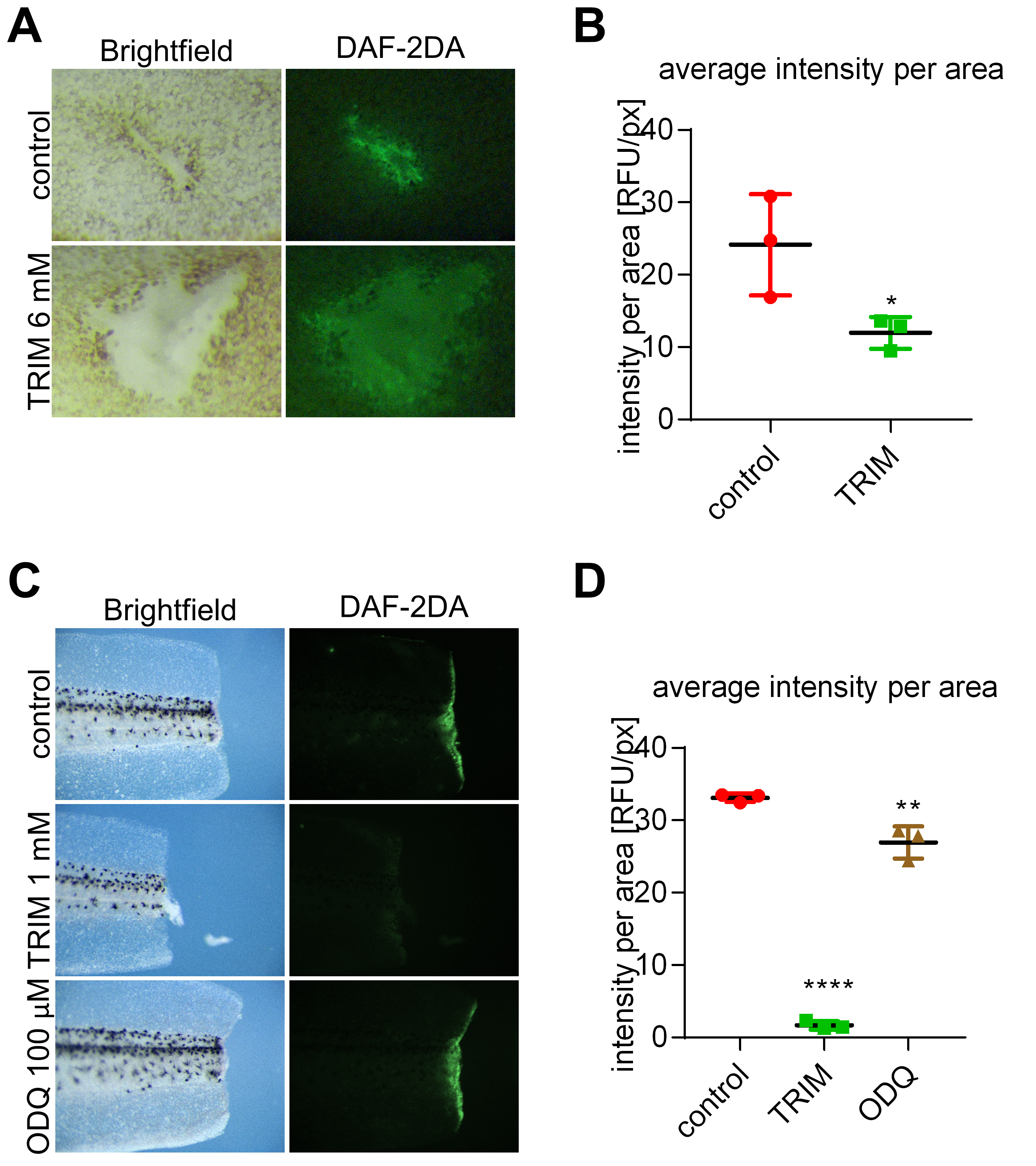

Supplement: Supplementary file 13 — Additional file 13. Figure S9 Test of the effectivity of TRIM to inhibit the production of NO and for the specificity of DAF-2DA to NO. (A) Control embryos at stage 26, embryos with inhibited production of NO using TRIM (6 mM) 1 hour before injury were injured using a needle, incubated in media with DAF-2DA solution for 10 minutes, fixed and imaged. (B) The intensity of signal were analysed (t-test). (C) Similarly, tails of tadpoles at stage 41, tadpoles with inhibited production of NO using TRIM (1 mM) or tadpoles with inihibited sGC with ODQ (100 μM), were amputated and incubated in media with DAF-2DA solution for 15 minutes, fixed and imaged. (D) The intensity of signal were analysed (t-test). **** - p < .0001, ** - p < .01, * - p < .05 [file 12864_2019_6147_MOESM13_ESM.png]
